# Retrospective Study of Critically Ill COVID-19 Patients With and Without Extracorporeal Membrane Oxygenation Support in Wuhan, China

**DOI:** 10.3389/fmed.2021.659793

**Published:** 2021-10-12

**Authors:** Wei Cheng, Xu-Dong Ma, Long-Xiang Su, Yun Long, Da-Wei Liu, Bin Du, Hai-Bo Qiu, Xiang-Dong Guan, De-Chang Chen, Yan Kang, Zhao-Hui Tong, Zhi-Yong Peng, You Shang, Rui-Qiang Zheng, Shu-Sheng Li, Chun Pan, Xiao-Bo Huang, Qing-Yuan Zhan, Ren-Yu Ding, Chao-Lin Huang, Yong-Jie Yin, Sheng-Qing Li, Xu-Yan Li, Li Jiang, Ming Hu, Xin Li, Xiang Zhou, Zhi-Cheng Jing, Yan-Hong Guo, Shu-Yang Zhang

**Affiliations:** ^1^Department of Critical Care Medicine, State Key Laboratory of Complex Severe and Rare Diseases, Peking Union Medical College Hospital, Chinese Academy of Medical Sciences, Beijing, China; ^2^Department of Medical Administration, National Health Commission of the People's Republic of China, Beijing, China; ^3^Department of Medical Critical Care Medicine, State Key Laboratory of Complex Severe and Rare Diseases, Peking Union Medical College Hospital, Chinese Academy of Medical Sciences, Beijing, China; ^4^Department of Critical Care Medicine, Zhongda Hospital, School of Medicine, Southeast University, Nanjing, China; ^5^Department of Critical Care Medicine, The First Affiliated Hospital of Sun Yat-sen University, Guangzhou, China; ^6^Department of Critical Care Medicine, Ruijin Hospital Affiliated to Medical College of Shanghai Jiaotong University, Shanghai, China; ^7^Department of Critical Care Medicine, West China Hospital of Sichuan University, Chengdu, China; ^8^Department of Respiratory and Critical Care Medicine, Beijing Institute of Respiratory Medicine, Beijing Chao-Yang Hospital, Capital Medical University, Beijing, China; ^9^Department of Critical Care Medicine, Zhongnan Hospital of Wuhan University, Wuhan, China; ^10^Department of Critical Care Medicine, Union Hospital, Tongji Medical College, Huazhong University of Science and Technology, Wuhan, China; ^11^Department of Critical Care Medicine, Northern Jiangsu People's Hospital, Yangzhou, China; ^12^Department of Critical Care Medicine, Tongji Hospital Affiliated to Tongji Medical College Huazhong University of Science and Technology, Wuhan, China; ^13^Department of Critical Care Medicine, Sichuan Academy of Medical Sciences & Sichuan Provincial People's Hospital, Chongqing, China; ^14^Department of Respiratory and Critical Care Medicine, China-Japan Friendship Hospital, Beijing, China; ^15^Department of Critical Care Medicine, The First Hospital of China Medical University, Shenyang, China; ^16^Department of Thoracic Surgery, Wuhan Jinyintan Hospital, Wuhan, China; ^17^Department of Emergency and Critical Care Medicine, The Second Hospital of Jilin University, Changchun, China; ^18^Department of Pulmonary and Critical Care Medicine, Huashan Hospital Affiliated to Fudan University, Shanghai, China; ^19^Department of Critical Care Medicine, Xuanwu Hospital Capital Medical University, Beijing, China; ^20^Department of Critical Care Medicine, Wuhan Pulmonary Hospital, Wuhan, China; ^21^Department of Cardiovascular Surgery, Zhongshan Hospital, Fudan University, Shanghai, China; ^22^Department of Cardiology, State Key Laboratory of Complex Severe and Rare Diseases, Peking Union Medical College Hospital, Chinese Academy of Medical Sciences, Beijing, China

**Keywords:** COVID-19, critically ill pneumonia, extracorporeal membrane oxygenation, in-hospital mortality, SARS-CoV-2

## Abstract

**Background:** Extracorporeal membrane oxygenation (ECMO) might benefit critically ill COVID-19 patients. But the considerations besides indications guiding ECMO initiation under extreme pressure during the COVID-19 epidemic was not clear. We aimed to analyze the clinical characteristics and in-hospital mortality of severe critically ill COVID-19 patients supported with ECMO and without ECMO, exploring potential parameters for guiding the initiation during the COVID-19 epidemic.

**Methods:** Observational cohort study of all the critically ill patients indicated for ECMO support from January 1 to May 1, 2020, in all 62 authorized hospitals in Wuhan, China.

**Results:** Among the 168 patients enrolled, 74 patients actually received ECMO support and 94 not were analyzed. The in-hospital mortality of the ECMO supported patients was significantly lower than non-ECMO ones (71.6 vs. 85.1%, *P* = 0.033), but the role of ECMO was affected by patients' age (Logistic regression OR 0.62, *P* = 0.24). As for the ECMO patients, the median age was 58 (47–66) years old and 62.2% (46/74) were male. The 28-day, 60-day, and 90-day mortality of these ECMO supported patients were 32.4, 68.9, and 74.3% respectively. Patients survived to discharge were younger (49 vs. 62 years, *P* = 0.042), demonstrated higher lymphocyte count (886 vs. 638 cells/uL, *P* = 0.022), and better CO_2_ removal (PaCO2 immediately after ECMO initiation 39.7 vs. 46.9 mmHg, *P* = 0.041). Age was an independent risk factor for in-hospital mortality of the ECMO supported patients, and a cutoff age of 51 years enabled prediction of in-hospital mortality with a sensitivity of 84.3% and specificity of 55%. The surviving ECMO supported patients had longer ICU and hospital stays (26 vs. 18 days, *P* = 0.018; 49 vs. 29 days, *P* = 0.001 respectively), and ECMO procedure was widely carried out after the supplement of medical resources after February 15 (67.6%, 50/74).

**Conclusions:** ECMO might be a benefit for severe critically ill COVID-19 patients at the early stage of epidemic, although the in-hospital mortality was still high. To initiate ECMO therapy under tremendous pressure, patients' age, lymphocyte count, and adequacy of medical resources should be fully considered.

## Background

In 2019, an epidemic of SARS-CoV-2 broke out in Hubei Province, China. Mortality of critically ill patients with mechanical ventilation was as high as 81% (1, 2). In a study of the influenza A/H1N1/2009 virus (3), extracorporeal membrane oxygenation (ECMO) was found to improve gas exchange, and subsequent randomized controlled studies (4, 5) also found positive effects of ECMO in severe ARDS patients. As for critically ill COVID-19 patients, ECMO might play its role.

To date, some studies reported the use of ECMO in the treatment of COVID-19 patients (6). Yang et al. (7) found that EMCO might be effective for these patients, but the sample size was small. Schmidt et al. (8) found the estimated 60-day survival of these patients were similar to that of recent studies (5, 9) and ECMO was recommended for severe ARDS associated with COVID-19 patients. However, several important key factors need to be mentioned. First, this study was performed in the largest ECMO center in Paris. All the medical care patients received was homogeneous. As ELSO illustrated, almost all the ECMO centers, especially the ones with platinum or gold levels, were located in North America and Europe. The experience learned there might not be applicable to other countries. And multicenter performance was an inevitable choice during the COVID-19 epidemic. Second, their patients were enrolled from March to May, when medical resources were in plenty. As WHO showed, the global health system faced tremendous pressure under local pandemics, and indications and contraindications of ECMO were not the only issues we considered, which will be very similar to what we experienced in Wuhan, China. Winter is coming, and another round of outbreak might be approaching. It is quite necessary for us to summarize the characteristics and outcome of these patients indicated for ECMO support to clarify its role and try to explore potential parameters for guiding the initiation during the COVID-19 epidemic.

## Methods

All confirmed COVID-19 patients admitted to 62 authorized hospitals in Wuhan from January 1, 2020, to May 1, 2020, were examined (10). Severe and critically ill patients supported with invasive mechanical ventilation were then enrolled. According to ***the diagnosis and treatment protocol for Novel Coronavirus Pneumonia of the National Health Commission of the People's Republic of China (version1)*** (Appendix 1), the patients who met the indications and had no contraindications of ECMO use were enrolled in this study. This study was approved by the SARS-CoV-2 Real World Research Program of Ministry of Science and Technology of China (S-K1297). This study only analyzed pre-existing non-identified data from a national registry; thus, no patient written informed consent was required to participate in this study in accordance with the national legislation and institutional requirements.

According to the protocol, indications for ECMO were as follows. Under optimal ventilation conditions (FiO_2_ ≥ 0.8, tidal volume = 6 ml/kg ideal weight, PEEP ≥10 cm H_2_O), if there was no contraindication, occurrence of one or more of the following conditions: (a) PaO_2_/FiO_2_ <50 mmHg for more than 3 h; (b) PaO_2_/FiO_2_ <80 mmHg for more than 6 h; (c) FiO_2_ = 1.0, PaO_2_/FiO_2_ <100 mmHg; (d) pH <7.25 and PaCO_2_ >60 mmHg for more than 6 h, with respiratory rate >35/min; (e) pH <7.2 and plateau pressure >30 cmH_2_O even respiratory rate >35/min; (f) severe air leakage syndrome. Contraindications to ECMO use were: (a) complicated with irreversible disease; (b) absolute contraindication of anticoagulation; (c) mechanical ventilation lasted for more than 7 days at higher ventilator settings (FiO2 > 0.9, Plateau pressure > 30 cmH2O); (d) vascular anatomical malformations or lesions in the puncture site; (e) advanced age; (f) immunosuppression (absolute neutrophil count <400/mm3).

After enrollment, the patients were divided into ECMO and non-ECMO groups according to whether ECMO was applied. Because of severe insufficiency of medical resources (ECMO devices or skilled personnel), not all the patients indicated received ECMO support. After retrieving medical records, we collected all information including gender, age, underlying comorbidities, vital signs and laboratory data at admission, treatment strategies (drugs and measurements), ECMO-related data (duration, flow, gas flow, FiO_2_, anticoagulation, and complications), duration of ICU and hospital stay, and in-hospital mortality. The survived patients were followed up to 90 days post ECMO weaning.

After outbreak of the COVID-19 pandemic, medical staff from all over the country aided Wuhan. On January 24, 2020, the first support arrived, and by February 15, 2020, all COVID-19 patients in Wuhan were “fully receivable” due to the arrival of many medical teams and the establishment of Huoshenshan, Leishenshan, and Fang Cang hospitals. According to these time points, we grouped the patients to identify the influence of medical equipment and skilled staff on the ECMO performance and in-hospital mortality.

### Statistical Analysis

Normally distributed data are expressed as the mean and standard deviation and compared using Students' *t*-test. Non-normally distributed data are presented as median and IQR and analyzed using Mann–Whitney *U*-test. Categorical variables are expressed as number and percentage and compared with the chi-square or Fisher's exact tests. Multivariate logistic regressions were performed successively to determine independent risk factors for in-hospital mortality. ROC analysis was performed to determine the discriminatory ability of parameters for predicting mortality. Youden's index was defined for points along the ROC curve, and the reliabilities were assessed by their sensitivity and specificity. All variables with *P* < 0.05 in the univariate analysis were considered significant. The results were expressed as *P*-value and odds ratio (OR) with 95% confidence interval (CI). IBM SPSS 23.0 software was used for all statistical analyses (IBM Corp., Armonk, NY).

## Results

**Figure 1:**
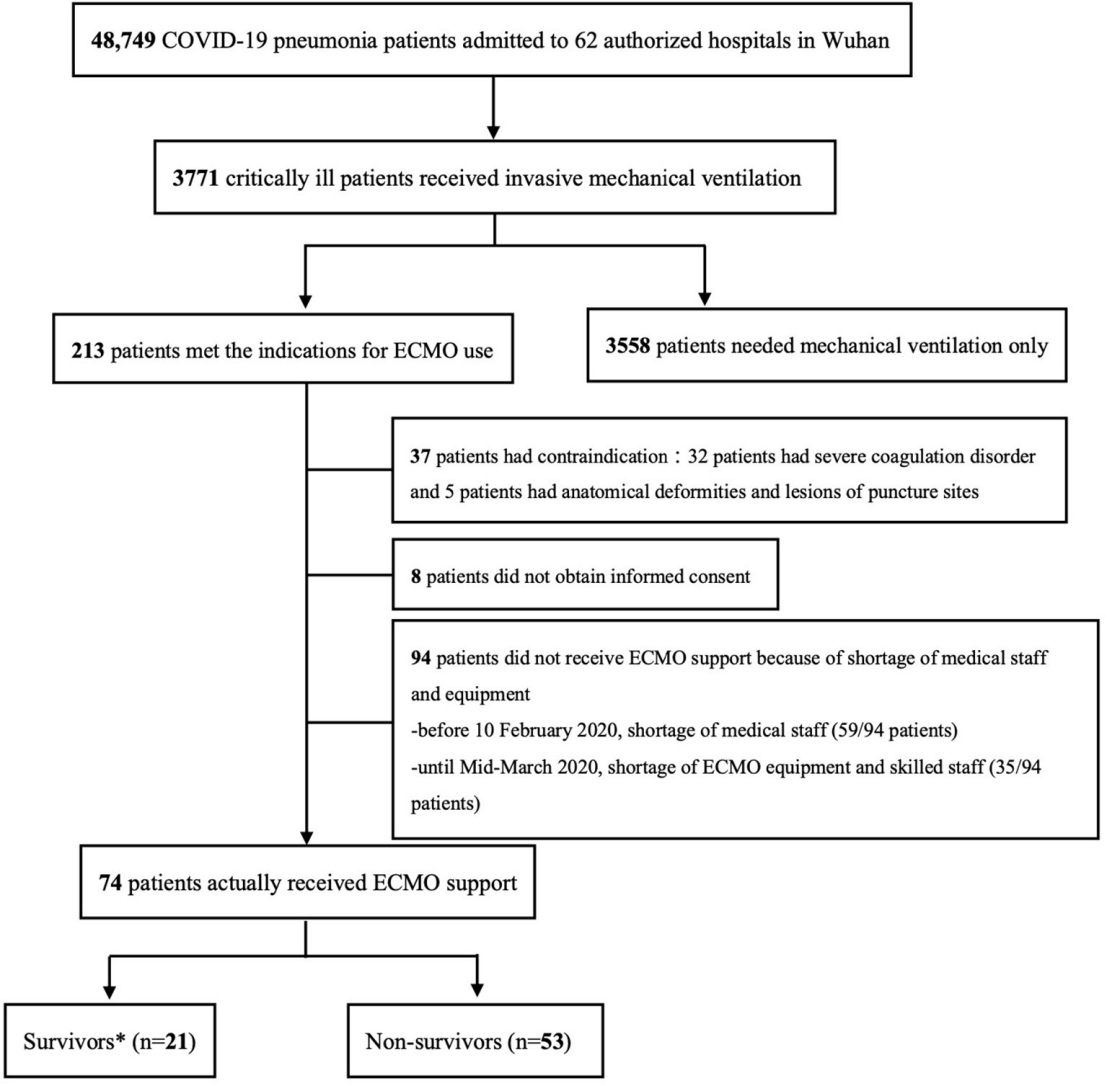
Enrollment Flowchart. ^*^Survivors and non-survivors were divided according to in-hospital mortality.

A total of 48,749 COVID-19 patients were admitted to the 62 authorized hospitals in Wuhan during the study period, and 3,771 patients were supported with invasive mechanical ventilation. Among the ventilated patients, excluding those with contraindications, 168 patients were included in this study, and 74 of them (44%) received ECMO support, while 94 patients were treated with mechanical ventilation only because of inadequacy of medical resources (Figure 1; Supplementary Tables 5–6). All the COVID-19 patients treated with ECMO in Wuhan were included in the study.

### Comparison Between ECMO Supported and Non-ECMO Patients

The whole patient cohort had a median age of 63 years (IQR 55–71 years), with 62.5% (105/168) being male. The patients receiving ECMO support had significantly lower in-hospital mortality than the control group (71.6 vs. 85.1%, *P* = 0.033), but significantly longer hospital and ICU stays (*P* = 0.033 and *P* = 0.006, respectively), significantly lower age (58 vs. 66, *P* < 0.0001), and more severe CO_2_ retention and acidosis at admission (*P* < 0.0001 and *P* = 0.015, respectively). Greater use of cortical steroids and tocilizumab was observed in the ECMO group, as they had higher plasma IL-6 concentration (319.7 ± 705.2 vs. 58.8 ± 79.5, *P* = 0.008). The proportion of prone position during ECMO support (39.2 vs. 21.3%, *P* = 0.011), vasoactive drug usage (97.3 vs. 80.9%, *P* = 0.016), and inter-hospital transfer with ECMO (41.9 vs. 24.5%, *P* = 0.016) were also higher in the ECMO group, as was the incidence of bacterial co-infection (45.9 vs. 27.7%, *P* = 0.014) (Table 1).

**Table 1:** Comparison between ECMO and non-ECMO patients.

	**Total**	**ECMO (*n* = 74)**	**Non-ECMO (*n* = 94)**	*P* ^*^
**Baseline characteristics**				
Gender (male %)	105 (62.5%)	46 (62.2%)	59 (62.8%)	0.936
Age (years) (M.IQR)	63 (55–71)	58 (47–66)	66 (60–76)	<0.0001
**Comorbidities**				
Hypertension	78 (46.4%)	30 (40.5%)	48 (51.1%)	0.175
Diabetes mellitus	40 (23.8%)	22 (29.7%)	18 (19.1%)	0.110
Cardiovascular disease	36 (21.4%)	21 (28.4%)	15 (16%)	0.051
Chronic pulmonary disease	8 (4.8%)	2 (2.7%)	6 (6.4%)	0.266
Chronic kidney disease	12 (7.1%)	8 (10.8%)	4 (4.3%)	0.101
Chronic liver disease	13 (7.7%)	8 (10.8%)	5 (5.3%)	0.186
Digestive disease	5 (3%)	1 (1.4%)	4 (4.3%)	0.271
Cerebral vascular disease	13 (7.7%)	7 (9.5%)	6 (6.4%)	0.459
Autoimmune and hematopathy	4 (2.4%)	0	4 (4.3%)	0.072
Solid tumor	9 (5.4%)	2 (2.7%)	7 (7.4%)	0.175
Time from onset to admission (days)	11 (7–18.5)	10 (6–17.25)	11 (7–20)	0.154
SOFA score	8 (7–9)	8 (6.75–9)	8 (6.5–9.5)	0.454
**Vital signs at admission**				
Heart rate (beats per minute)	93 ± 19	95 ± 23	91 ± 16	0.175
Temperature (°C)	36.9 ± 0.9	36.9 ± 0.9	36.9 ± 0.9	0.885
Systolic Blood Pressure (mmHg)	125 ± 21	123 ± 23	127 ± 19	0.236
Diastolic Blood Pressure (mmHg)	74 ± 13	72 ± 14	75 ± 12	0.133
Respiratory rate (beats per minute)	23 ± 6	23 ± 6	22 ± 5	0.106
**Laboratory results at admission**				
White Blood Cell (^*^109/L)	10.8 ± 6.3	11.9 ± 6.2	9.9 ± 6.3	0.054
Neutrophil (^*^109/L)	9.59 ± 5.84	10.3 ± 5.9	8.7 ± 5.6	0.124
Lymphocyte (cells/dL)	710 ± 490	710 ± 410	710 ± 560	0.959
Lactate (mmol/L)	2.8 ± 2.3	3.3 ± 2.9	2.1 ± 1.1	0.073
Platelet (^*^109/L)	129.8 ± 105.7	160 ± 93.6	194 ± 112	0.055
Total Bilirubin (umol/L)	17.6 ± 12.5	19.8 ± 15.6	15.8 ± 8.9	0.086
Creatinine (umol/L)	89.7 ± 82	77.1 ± 42.5	98.6 ± 100	0.143
High sensitivity C-reactive protein (mg/L)	83 ± 72.7	78.6 ± 81	87 ± 64	0.524
Erythrocyte Sedimentation Rate (mm/H)	57.1 ± 35.3	60.4 ± 35.5	55.6 ± 35.8	0.592
Ferritin (ng/ml)	1092 ± 1139	1668 ± 1819	861 ± 636	0.057
Procalcitonin (ng/ml)	2.79 ± 7.97	3.27 ± 8.43	2.38 ± 7.59	0.518
(1,3) - β - D-glucan (pg/ml)	47.6 ± 38.4	48.6 ± 33	47.4 ± 39.8	0.939
Interleukin-6 (pg/ml)	172.9 ± 484.9	319.7 ± 705.2	58.8 ± 79.5	0.008
Interleukin-8 (pg/ml)	49.6 ± 42.3	44.7 ± 41.7	55.9 ± 45.6	0.617
**Respiratory parameters**				
**At admission**				
PaO2 (mmHg)	81.5 ± 52.5	89.5 ± 45.7	74.5 ± 57.2	0.128
FiO2 (%)	65.5 ± 23.5	67 ± 25	64 ± 22	0.632
PaCO2 (mmHg)	42.5 ± 18.7	49.3 ± 17.1	36.6 ± 18.1	<0.0001
PH value	7.33 ± 0.42	7.22 ± 0.59	7.42 ± 0.09	0.015
**Before intubation**				
PaO2 (mmHg)	71.9 ± 40.3	82.1 ± 42.1	58.1 ± 33.7	0.007
PaCO2 (mmHg)	46.9 ± 21.1	52.5 ± 18	40 ± 22.8	0.006
Time from severe ARDS to intubation (days)	1 (1–4)	1 (1–6.25)	1 (1–2.5)	0.129
**Treatment strategies**				
Inter-hospital Transfer	54 (32.1%)	31 (41.9%)	23 (24.5%)	0.016
Vasoactive drugs	148 (88.1%)	72 (97.3%)	76 (80.9%)	0.001
Anti-viral drugs	79 (47%)	34 (45.9%)	45 (47.9%)	0.804
Cortical steroids	142 (84.5%)	68 (91.9%)	74 (78.7%)	0.019
Tocilizumab	7 (4.2%)	7 (9.5%)	0	0.002
Prone position	49 (29.2%)	29 (39.2%)	20 (21.3%)	0.011
**Prognosis related parameters**				
Co-infection of bacteria	60 (35.7%)	34 (45.9%)	26 (27.7%)	0.014
ICU stays (days)	18 (10–30.5)	21 (12.75–33)	15 (5–30)	0.033
Hospital stays (days)	27 (12–39.75)	32 (16–45.5)	21.5 (12–34)	0.006
In-hospital Mortality	133 (79.2%)	53 (71.6%)	80 (85.1%)	0.033

*M, mean; IQR, interquartile range; PaO2, partial pressure of oxygen; FiO2, fraction of inspired oxygen; PaCO2, partial pressure of carbon dioxide; ARDS, acute respiratory distress syndrome; ICU, intensive care unit*.

* *P-value for the comparison between ECMO and non-ECMO group*.

### Comparison Between Survivors and Non-survivors of all the Enrolled Patients

The overall in-hospital mortality of the whole population was 79.2%. Upon dividing the patients into survival and non-survival groups, it was apparent that the patients in the survival group were significantly younger (56 vs. 64 years, *P* = 0.001) with a higher ECMO application rate (60 vs. 39.8%, *P* = 0.033), but longer ICU and hospital stays (Supplementary Table 7). After multivariate logistic regression, ECMO application was not approved to be an independent risk factor for in-hospital mortality [OR 0.62, 95% CI (0.275, 1.383), *P* = 0.240] (Supplementary Table 8).

### Characters of the ECMO Supported Patients

Patients supported with ECMO had a median age of 58 years, with 62.2% being male. The in-hospital mortality of these patients was 71.6%. At 90 days after ECMO weaning, the 28-, 60-, and 90-day mortalities were 32.4, 68.9, and 74.3% respectively (Supplementary Figure 3). The median time from disease onset to admission was 10 days, and the median ICU and hospital stays of the patients were 21 and 32 days, respectively. The patients survived to discharge demonstrated lower age (49 vs. 62 years, *P* = 0.042), higher lymphocyte counts at admission (886 vs. 638 cells/uL, *P* = 0.022), but longer ICU and hospital stays. There were no significant differences in comorbidities, vital signs, and other laboratory results at admission between the survivors and non-survivors according to in-hospital mortality (Table 2).

**Table 2:** Clinical characters of the ECMO-supported patients.

	**ALL ECMO**	**Survivors (*n* = 21)**	**Non-survivors (*n* = 53)**	*P* ^*^
**Baseline characteristics**				
Sex (male %)	46 (62.2%)	14 (66.7%)	32 (60.4%)	0.615
Age (years) (M,IQR)	58 (47–66)	49 (42–62)	62 (54–68)	0.042
**Comorbidities**				
Hypertension	30 (40.5%)	10 (47.6%)	20 (37.7%)	0.435
Diabetes Mellitus	22 (29.7%)	7 (33.3%)	15 (28.3%)	0.669
Cardiovascular disease	21 (28.4%)	4 (19%)	17 (32.1%)	0.262
Chronic pulmonary disease	2 (2.7%)	0	2 (3.8%)	0.367
Chronic kidney disease	8 (10.8%)	3 (14.3%)	5 (9.4%)	0.545
Chronic liver disease	8 (10.8%)	0	8 (15.1%)	0.059
Digestive disease	1 (1.4%)	0	1 (1.9%)	0.526
Cerebral vascular disease	7 (9.5%)	2 (9.5%)	5 (9.4%)	0.991
Autoimmune and hematopathy	0	0	0	1.000
Solid tumor	2 (2.7%)	0	2 (3.8%)	0.367
Time from onset to admission (days)	10 (6–17.25)	12 (5.5–17)	10 (6–18)	0.684
SOFA score	8 (6.75–9)	9 (7–9.5)	8 (6–9)	0.145
**Vital signs at admission**				
Heart rate (beats per minute)	95 ± 23	97 ± 22	94 ± 23	0.631
Temperature (°C)	36.9 ± 0.9	36.9 ± 1.0	36.9 ± 0.9	0.801
Systolic Blood Pressure (mmHg)	123 ± 23	119 ± 22	125 ± 24	0.370
Diastolic Blood Pressure (mmHg)	72 ± 14	72 ± 14	72 ± 13	0.981
Respiratory Rate (beats per minute)	23 ± 6	24 ± 6	23 ± 7	0.944
**Laboratory results at admission**				
White Blood Cell (^*^109/L)	11.9 ± 6.2	12.7 ± 7.5	11.6 ± 5.7	0.489
Neutrophil (^*^109/L)	10.3 ± 5.98	9.92 ± 6.67	10.4 ± 5.76	0.744
Lymphocyte (cells/uL)	710 ± 410	886 ± 537	638 ± 335	0.022
Lactate (mmol/L)	3.33 ± 2.91	2.75 ± 1.89	3.57 ± 3.26	0.542
Platelet (^*^109/L)	160 ± 93.7	154 ± 61.5	162 ± 105.6	0.738
Total Bilirubin (umol/L)	19.8 ± 15.6	15.9 ± 10.7	21.4 ± 17.1	0.257
Creatinine (umol/L)	77.1 ± 42.5	79.3 ± 51.5	76.1 ± 38.7	0.809
High sensitivity C-reactive protein (mg/L)	78.6 ± 81	65.2 ± 51.2	83.5 ± 89.6	0.458
Erythrocyte Sedimentation Rate (mm/H)	60.4 ± 35.5	51.3 ± 26.2	66.3 ± 40.1	0.336
Ferritin (ng/ml)	1669 ± 1819	846.8 ± 1140	2901 ± 2095	0.077
Procalcitonin (ng/ml)	3.3 ± 8.4	1.81 ± 4.36	3.89 ± 9.66	0.371
(1,3) - β - D-glucan (pg/ml)	48.6 ± 33.0	62.9 ± 43.9	37.9 ± 23.2	0.370
Interleukin-6 (pg/ml)	319.7 ± 705.2	468 ± 665	237 ± 725	0.315
Interleukin-8 (pg/ml)	44.7 ± 41.7	53.2 ± 51.6	34 ± 28.4	0.529
PaO2 (mmHg)	89.5 ± 45.7	93.9 ± 50.2	88.1 ± 44.7	0.691
PaCO2 (mmHg)	49.3 ± 17.1	45.3 ± 15.8	50.7 ± 17.5	0.327
PH value	7.22 ± 0.59	7.06 ± 0.71	7.29 ± 0.54	0.234
**Prognosis related parameters**				
ICU stays (days)	21 (12.75–33)	26 (17.5–38)	18 (10.5–30)	0.018
Hospital stays (days)	32 (16–45.5)	49 (35.5–74)	29 (13.5–37)	<0.001

*ECMO, extracorporeal membrane oxygenation; PaO2, partial pressure of oxygen; PaCO2, partial pressure of carbon dioxide; ARDS, acute respiratory distress syndrome; FiO2, fraction of inspired oxygen; ICU, intensive care unit*.

* *P-value for the comparison between survivors and non-survivors according to in-hospital mortality*.

The median time from intubation to ECMO initiation was 3.5 days, but the median time from severe ARDS to ECMO initiation was 7 days. The median duration of ECMO support was 13 days, with an ECMO rotation of 3069 rotate per minute and a blood flow of 3.7 L/min at 1st day of ECMO support. Treatment of ECMO with prone position was used in 39.2% of the patients. Bleeding complications occurred in 48.6% of patients, whilst bacterial co-infection was observed in 39.2% of the patients. Successful weaning of ECMO was achieved in 29 patients (39.2%). There were no significant differences in ventilation parameters before ECMO between survivors and non-survivors according to in-hospital mortality. Following ECMO treatment, COVID-19 patients survived to discharge had lower CO_2_ levels after ECMO initiation (39.7 vs. 46.9, *P* = 0.041) but higher risk of co-infections (57.1 vs. 32.1%, *P* = 0.046) (Table 3).

**Table 3:** Variables associated with ECMO treatment.

	**ALL ECMO**	**Survivors (*n* = 21)**	**Non-survivors (*n* = 53)**	*P* ^*^
**Ventilation parameters before ECMO**				
FiO2 (%)	85 (80–96)	80 (80–90)	90 (80–90)	0.193
PEEP (cmH2O)	15 (12–16.5)	14 (12–15.5)	15 (13–18)	0.224
Tidal Volume (mL/kg predicted bodyweight)	5 (5–6)	5 (5–6)	5 (5–6)	0.281
Pplateau (mmHg)	34 (32–35)	33 (30–35)	34 (32–35)	0.601
RR (per minute)	30 (28–32)	30 (28–32)	30 (28–32)	0.451
PaO2-Pre ECMO^#^ (mmHg)	82.1 ± 42.1	92.1 ± 63.6	79.1 ± 33.9	0.374
PaCO2-Pre ECMO^#^ (mmHg)	52.5 ± 18.0	45.5 ± 18.5	54.4 ± 17.7	0.166
**ECMO Treatment-related parameters**				
Time from severe ARDS to intubation(days)	1 (1–6.25)	1(1–7.25)	1 (1–6)	0.444
Time from intubation to ECMO initiation(days)	3.5(2–8.75)	2(1–8)	5 (2–9.5)	0.588
Time from severe ARDS to ECMO initiate(days)	7 (2–14)	5(2–13)	7.5(3–15)	0.725
Duration of ECMO (days)	13(8–21)	9(6.5–15.8)	15(9–22)	0.192
ECMO rotation at D1(rotate per minute)	3069 ± 538	3158 ± 386	3025 ± 600	0.399
ECMO blood flow at D1(Liter per minute)	3.7 ± 0.8	3.6 ± 0.3	3.7 ± 0.9	0.698
ECMO gas flow at D1(L/min)	4.8 ± 1.7	4.6 ± 1.3	4.9 ± 1.9	0.704
ECMO FiO2 at D1 (%)	67.5 ± 25.9	61 ± 24.5	70 ± 26.5	0.251
**Activated Partial Thromboplastin Time (s)**				
Day 1 after ECMO initiation	51.1 ± 24.2	52.3 ± 27.3	50.5 ± 23.1	0.806
Day 3 after ECMO initiation	58.3 ± 28.6	67.7 ± 42.5	53.7 ± 17.7	0.108
Day 7 after ECMO initiation	54.9 ± 17.4	51.9 ± 16.1	56.7 ± 18.1	0.382
**Complication-related parameters of ECMO**				
**Bleeding complications**				
Total	36(48.6%)	9(42.9%)	27(50.9%)	0.530
Gastrointestinal Bleeding	25(33.8%)	8(38.1%)	17(32.1%)	0.622
Incision bleeding	6 (8.1%)	2(9.5%)	4 (7.5%)	0.779
Airway Bleeding	12(16.2%)	2(9.5%)	10(18.9%)	0.326
Hemorrhagic shock	29(39.2%)	7(33.3%)	22(41.5%)	0.516
**Transfusion**				
Total (ml)	5053 ± 6694	6088 ± 7638	4679 ± 6367	0.462
Red Blood Cell (ml)	2725 ± 2715	3550 ± 2622	2473 ± 2721	0.197
**Co-infection of bacteria**				
Any	29(39.2%)	12(57.1%)	17(32.1%)	0.046
Incision site infection	2 (2.7%)	2 (9.5%)	0	0.023
Blood stream infection	13(17.6%)	6 (28.6%)	7 (13.2%)	0.117
Pulmonary bacterial infection	28(37.8%)	12(57.1%)	16(30.2%)	0.031
**Mechanical complication**				
Obstruction	2(2.7%)	0	2(3.8%)	0.367
Hemolysis	7(9.5%)	4(19%)	3(5.7%)	0.076
prolapse	1(1.4%)	1(4.8%)	0	0.110
**Reasons for ECMO withdrawing**				
Planned	17(23%)	15(71.4%)	2 (3.8%)	0.0001
Complication	12(16.2%)	6 (28.6%)	6 (11.3%)	
Death	45(60.8%)	0	45(84.9%)	
**Weaning success**				
Yes	29(39.2%)	21(100%)	8 (15.1%)	<0.0001
No	45(60.8%)	0	45(84.9%)	
**Ventilation parameters after ECMO**				
FiO2 (%)	40 (35–45)	40 (35–45)	40 (40–45)	0.217
PEEP (cmH2O)	8 (8–10)	8 (7–10)	8 (8–10)	0.403
Tidal Volume (mL/kg predicted bodyweight)	4 (4–5)	4 (3.8–4.8)	4 (4–5)	0.950
Pplateau (mmHg)	26 (25–28)	26 (25–28)	26 (25–28)	0.891
RR (per minute)	15 (12–16.5)	15 (13–16)	15 (12–18)	0.733
PaO2 post-ECMO^&^ (mmHg)	103.5 ± 58.5	121.8 ± 60.2	96.8 ± 57.4	0.210
PaCO2 post-ECMO^&^ (mmHg)	45.0 ± 13.2	39.7 ± 8.0	46.9 ± 14.3	0.041
**Other Treatment strategies**				
Prone position plus ECMO	29(39.2%)	8(38.1%)	21(39.6%)	0.903
Inter-hospital transfer with ECMO	31(41.9%)	8(38.1%)	23(43.4%)	0.677
vasoactive drugs	72(97.3%)	20(95.2%)	53(100%)	0.110
anti-viral drugs	34(45.9%)	8(38.1%)	26(49.1%)	0.394
cortical steroids	68(91.9%)	20(95.2%)	48(90.6%)	0.507
Tocilizumab	7 (9.5%)	2 (9.5%)	5 (9.4%)	0.991

*ECMO, extracorporeal membrane oxygenation; PaO2, partial pressure of oxygen; PaCO2, partial pressure of carbon dioxide; ARDS, acute respiratory distress syndrome; FiO2, fraction of inspired oxygen; ICU, intensive care unit*.

# *the last data pre-ECMO; & the first data post ECMO*.

* *P value for the comparison between survivors and non-survivors according to in-hospital mortality*.

### Predictors of In-Hospital Mortality for ECMO Patients

Multivariate logistic regression showed that age [OR 1.14, 95% CI (1.027, 1.254), *P* = 0.013] and CO_2_ level after ECMO initiation [OR 1.17, 95% CI (1.038, 1.309), *P* = 0.01] were independent risk factors for in-hospital mortality (Table 4).

**Table 4:** Multivariate Logistic regression analysis of variables affecting prognosis of ECMO patients.

	**SE**	**Wald**	**OR**	**95%CI**	***P*-value**
Age	0.051	6.112	1.14	1.027, 1.254	0.013
Lymphocyte	1.637	3.005	0.06	0.002, 1.449	0.083
Co-infection	1.083	2.453	0.18	0.022, 1.532	0.117
PaCO2 after ECMO	0.059	6.666	1.17	1.038, 1.309	0.01

*ECMO, extracorporeal membrane oxygenation; SE, stand error of mean; OR, odd ratio; CI, confidence interval; PaCO2 after ECMO, partial pressure of carbon dioxide immediately after ECMO initiation*.

Age was an independent risk factor for the prognosis of ECMO patients, and to assess its ability to predict prognosis, we performed ROC analyses of age and lymphocyte count. Age had better discriminatory ability. A cutoff age of 51 years enabled prediction of the in-hospital mortality of ECMO patients with a sensitivity of 84.3% and specificity of 55% (*P* = 0.038; Supplementary Figure 4). To further illustrate the role of age in the ECMO supported patients, we divided the patients into two groups. The patients aged <51 years got lower in-hospital mortality (42.9 vs. 83%, *P* = 0.001) and higher success of ECMO weaning (61.9 vs. 30.2%, *P* = 0.012) (Supplementary Table 9).

**Figure 2:**
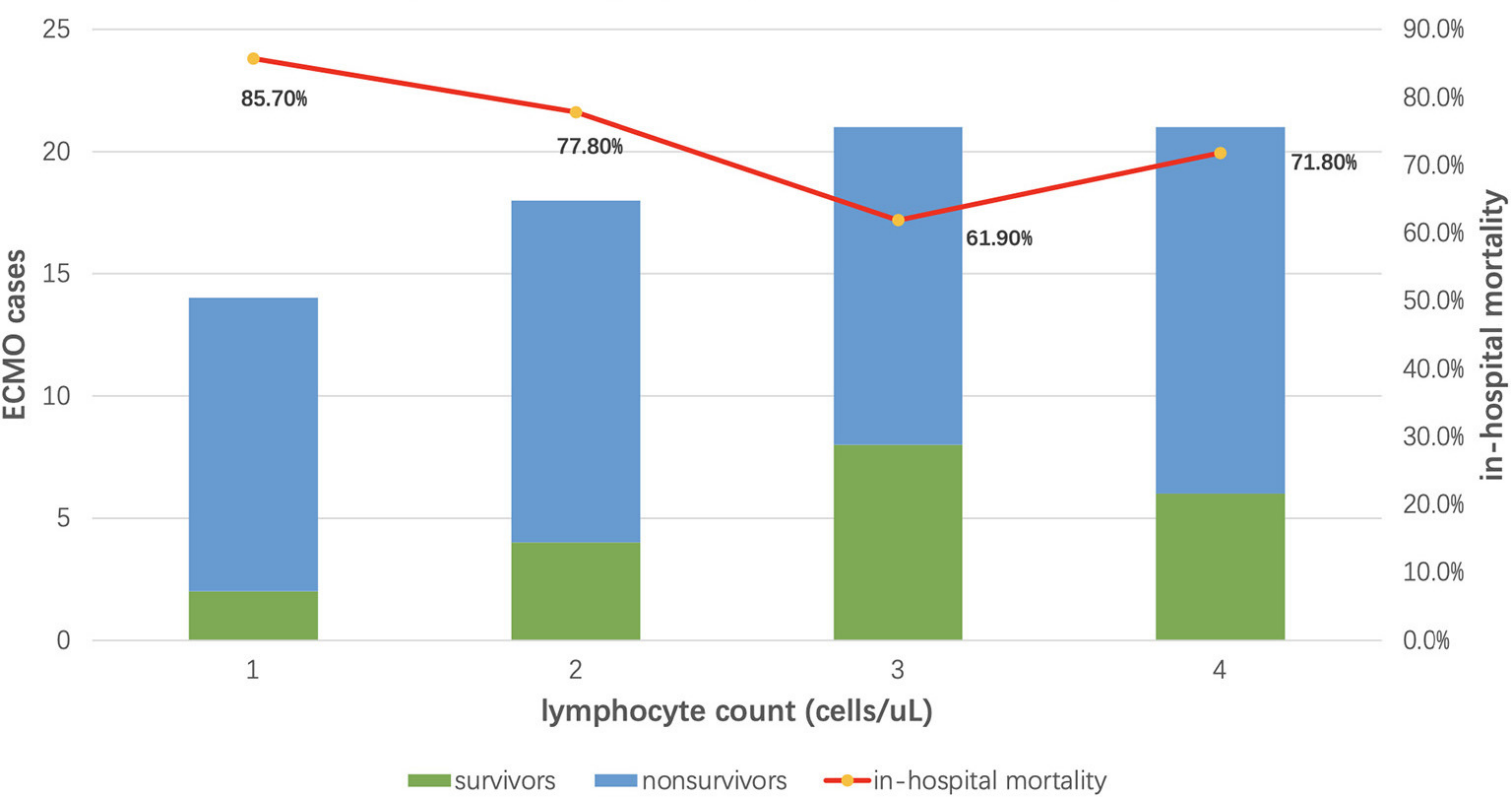
The relationship between lymphocyte count and in-hospital mortality. The median lymphocyte counts of all the enrolled patients were 590 cells/uL. According to IQR, ECMO patients were divided into four groups. The in-hospital mortality of patients with higher lymphocyte counts was lower (in-hospital mortality from group 1 to 4: 85.7, 77.8, 61.9, and 71.8%).

The median lymphocyte counts of all the enrolled patients were 590 cells/uL. According to IQR, ECMO patients were divided into four groups. The in-hospital mortality of patients with higher lymphocyte counts was lower (in-hospital mortality from group 1 to 4: 85.7, 77.8, 61.9, and 71.8%) (Figure 2). Additionally, we also found that plasma IL-6 concentration and neutrophil-to-lymphocyte ratio (NLR) in ECMO patients were also slightly correlated with patients' in-hospital mortality (Supplementary Figures 5, 6).

After outbreak of COVID-19 in Wuhan, the health system was under tremendous pressure. The first ECMO procedure on COVID-19 patients was performed on January 7, 2020, and 4 days later the patient died. Not until February 15, 2020, when medical staff and resources were fully supplemented, only 24 critically ill patients received ECMO, whilst 59 others only received mechanical ventilation. Patients with ECMO were divided into two groups according to this date. Many more ECMO cases (50/74) were performed after February 15, 2020, and patients were slightly more severe with higher SOFA score (8 vs. 9, *P* = 0.145) and more comorbidities. More intensive procedures like prone position (46 vs. 25%, *P* = 0.083) and inter-hospital transfer with ECMO (48 vs. 29.2%, *P* = 0.124) were taken. However, many more complications occurred (bleeding 60 vs. 25%, *P* = 0.005; bacterial co-infection rate, 50 vs. 16.7%, *P* = 0.006). The in-hospital mortality was slightly decreased (75 vs. 70%) but the hospital stay was significantly prolonged (18.5 vs. 36.5 days, *P* = 0.016) (Supplementary Table 10).

## Discussion

This is a multi-center retrospective study to give detailed description of critically ill COVID-19 patients who needed ECMO support in China at the early stage of the epidemic. It clearly showed that certain amount of critically ill patients failed to receive ECMO support, and many more factors need to be considered under such extreme pressure, especially in unmatured ECMO centers. Our analyses showed that age was an independent predictor for in-hospital mortality, lymphocyte count, and adequacy of medical resources also showed an association with in-hospital mortality, which taken together provided additional information for the first time to help clinicians make decisions regarding whether to initiate ECMO support at this critical period.

When, how, and to whom to initiate ECMO should be fully considered when resources are stretched. A nationwide retrospective study found that age influenced COVID-19 patients' prognosis (11). Our study also found that age was an independent risk factor affecting in-hospital mortality of ECMO supported patients. Ages might help in making difficult choices under tremendous pressure. A major clinical manifestation of COVID-19 was lymphocytosis, and the degree of reduction was related to severity of the disease and prognosis (1, 12, 13). The delicate immune status could affect the prognosis of patients if ECMO was started (14). This concern was confirmed in this study as the non-surviving COVID-19 patients with ECMO had significantly lower lymphocyte counts, which could also help in deciding whether to carry out this procedure. We also found an association between patient mortality and IL-6 levels as Ruan et al. illustrated (12). Furthermore, a correlation between NLR and patient prognosis was also identified, which had been shown to be correlated with the severity of ARDS (15). Under conditions of limited medical resources, integrating age, lymphocyte counts, IL-6, and NLR may be helpful to screen for ECMO applicability and ultimately improve patient outcomes.

Our research team recently found that the overall survival rate of ECMO patients in Mainland China was up to the level of developed countries (9). But overall in-hospital mortality of ECMO supported COVID-19 patients appeared considerably higher than that in a recent series by Schmidt, although 28-day mortality rate was similar (8). The possible reasons might be as follows: (1) This was not a multicenter study. There might be a broad variation in ECMO professional personnel, resource, and experience in the 11 enrolled hospitals of our study. The situation should be similar to that in most countries as almost all the ECMO centers were in North America and Europe. Multicenter perform was an inevitable choice during the COVID-19 epidemic. (2) Extreme pressure. In the early stage of epidemic, a large number of critically ill patients emerged in a short period of time. The medical resources were seriously relatively deficient; therefore, the patients might not be well-cared for. Yang et al. (1) found that the 28 days mortality of patients with invasive mechanical ventilation was up to 80%. Another round of outbreak might be approaching when winter is coming. The global health system will face tremendous pressure under local pandemic and potential parameters we explored for guiding the initiation of ECMO could be of great use. (3) Timing of ECMO initiation. The time from diagnosis of severe ARDS to initiation of ECMO in our study was 7 days, which was longer than 4 days in Schmidt's study. Early application of ECMO could be more effective for severe ARDS (4, 5). (4) Other factors. The patients in our study were much older than those in Schmidt's study (58 vs. 49 years); inter-hospital transfer of our patients with ventilator and ECMO was up to more than 40% in our study, as mortality might be up to 50% even in patients transferred on mechanical ventilation (16), but there was no expertise ECMO transportation team in China until now. As medical resources in most parts of the world are not comparable to those in France, our results in the early stage were of great value in the epidemic.

In addition, there are some avoidable factors that lead to higher in-hospital mortality in our study. First, there is a relatively high incidence of ECMO-related complications. Hemorrhage and hospital-acquired infections were the most frequent types, and complications could cause up to 10% of the mortality of ECMO supported patients (5, 17, 18). In our study, bleeding complications were as high as 48.6, and 39.2% progressed into hemorrhagic shock. The incidence of bacterial co-infection was 39.2%. Relatively high incidence of complications could actually increase in-hospital mortality. Second, the application of ECMO had selectivity bias. Due to scarcity of medical staff and resources, clinicians preferred to devote the limited resources and energy to younger patients who they believed could have better prognosis (19). We found that the median age of patients with ECMO support was 58 years old, which was significantly lower than that of the non-ECMO group, and these patients received much more aggressive treatment. However, we should note that the ECMO supported patients got more severe CO2 retention and acidosis, had higher plasma Il-6 concentrations, and had higher co-infection rate. This “prejudice” could mask the actual role of ECMO.

It was observed that the ICU and hospital stay in patients with ECMO were significantly longer, especially the patients who survived to discharge. As ECMO support is a technically demanding, high-risk, and high-cost operation, and it is not a therapy to be rushed to the frontline (20). Abundant staff, experienced ECMO teams, and a relative high number of ECMO machines are indispensable for the smooth development of ECMO (21). Due to the local COVID-19 epidemic in Hubei province and the subsequent coordination of the National Health Commission of China, a total of 255 medical teams from across the country with a total of 32,572 medical staff had been sent to support Wuhan. This greatly alleviated the medical pressure and made ECMO cases more likely to be performed and better carried out. Sometimes, for critically ill patients, skilled medical staff and spare ECMO machines were not available at the same time, and there were still patients, although the number had decreased, who needed ECMO support but were unable to receive it. From the time distribution of ECMO cases, the procedure was carried out more frequently, and patients with higher SOFA score and with more comorbidities received greater ECMO support; however, the survival rate was only slightly increased after February 15, 2020, when medical resources and skilled staff were fully supplemented. In general, the reservation and distribution of high-end devices quickly and flexibly in extreme conditions were difficult problems to be solved in the future.

## Limitations

There are several limitations to this study. First, the sample size was not big enough, although we included all the ECMO supported COVID-19 patients in Wuhan, and the conclusion was only for reference with more influencing factors to be considered according to the actual situation. Second, due to the selection bias in reality, the role of ECMO in COVID-19 patients was not clarified, and randomized controlled trials, which were very difficult to carry out during this unprecedented period, should have been performed to assess the effect of ECMO. Third, this is a retrospective observational study and observed differences may still be subject to unobserved confounding factors we could not control for in our analyses.

## Conclusions

The in-hospital mortality of ECMO supported critically ill COVID-19 patients was high but reduced compared to no ECMO support, and age was an independent risk factor for in-hospital mortality. To initiate ECMO therapy under this epidemic situation, patients' age, lymphocyte count, and adequacy of medical resources should be fully considered.

## Data Availability Statement

The original contributions presented in the study are included in the article/Supplementary Material, further inquiries can be directed to the corresponding author/s.

## Ethics Statement

The studies involving human participants were reviewed and approved by the SARS-CoV-2 Real World Research Program of Ministry of Science and Technology of China (S-K1297). This study only analyzed pre-existing non-identified data from a national registry, thus, patient written informed consent was not required to participate in this study in accordance with the national legislation and the institutional requirements. Written informed consent for participation was not required for this study in accordance with the national legislation and the institutional requirements.

## Author Contributions

WC, X-DM, and L-XS contributed to data collection, interpretation, and drafted the manuscript. XZ, Z-CJ, Y-HG, and S-YZ contributed to the conception of the study and critically revised the manuscript for important intellectual content. YL, D-WL, BD, H-BQ, X-DG, D-CC, YK, Z-HT, Z-YP, YS, R-QZ, S-SL, CP, X-BH, Q-YZ, R-YD, C-LH, Y-JY, S-QL, X-YL, LJ, MH, and XL contributed to data collection and interpretation. All authors contributed to the article and approved the submitted version.

## Funding

This study was supported by National Key R&D Program of China (grant number 2020YFC0861000) and CAMS Innovation Fund for Medical Sciences (CIFMS) (No. 2020-I2M-CoV19-001), Beijing Municipal Natural Science Foundation (M21019), and CMB Open Competition Program (20-381).

## Conflict of Interest

The authors declare that the research was conducted in the absence of any commercial or financial relationships that could be construed as a potential conflict of interest.

## Publisher's Note

All claims expressed in this article are solely those of the authors and do not necessarily represent those of their affiliated organizations, or those of the publisher, the editors and the reviewers. Any product that may be evaluated in this article, or claim that may be made by its manufacturer, is not guaranteed or endorsed by the publisher.

## Abbreviations

ECMO: extracorporeal membrane oxygenation
ICU: intensive care units
ARDS: acute respiratory distress syndrome
NLR: neutrophil-to-lymphocyte ratio
IL-6: interleukin 6
PaO2: partial pressure of oxygen
PaCO2: partial pressure of carbon dioxide
FiO2: fraction of inspired oxygen
OR: odd ratio
95% CI: 95% confidence interval
PaCO2 after ECMO: the first partial pressure of carbon dioxide after ECMO initiation.
