# Supplementary material for: Retrospective Study of Critically Ill COVID-19 Patients With and Without Extracorporeal Membrane Oxygenation Support in Wuhan, China
Source: Front Med (Lausanne). 2021 Oct 12;8:659793. doi: 10.3389/fmed.2021.659793 (PMC8546219; doi:10.3389/fmed.2021.659793)
Supplement: Supplementary file 1 [file Data_Sheet_1.zip › 20210120-appendix 1 Recommended treatment plan for NCP .pdf]

Recommended treatment plan for novel coronavirus pneumonia (NCP) in severe and critically ill patients (PUMCH criterion)

Xiang Zhou[1], Wei Cao[1], Zhenyin Liu[1], Jinglan Wang[1], Ding Han[1], Taisheng Li[1], Bin Du[1], Shuyang Zhang[1]

1Peking Union Medical College Hospital, Peking Union Medical College and Chinese Academy of Medical Sciences, Beijing 100730, China

Dr. Xiang Zhou and Dr. Wei Cao contributed equally to this work.

#Corresponding author:

Shuyang Zhang

Peking Union Medical College Hospital

Peking Union Medical College and Chinese Academy of Medical Sciences, Beijing 100730, China

Tel./Fax: +86-10-69152300

E-mail: shuyangzhang103@163.com.

OR

Bin Du

Peking Union Medical College Hospital

Peking Union Medical College and Chinese Academy of Medical Sciences, Beijing 100730, China

Tel./Fax: +86-10-69152300

E-mail: dubin98@gmail.com

Or

Taisheng Li

Peking Union Medical College Hospital

Peking Union Medical College and Chinese Academy of Medical Sciences, Beijing 100730, China

Tel./Fax: +86-10-69152300

E-mail: litsh@263.net

Since December 2019, new infectious diseases with lung lesions caused by the 2019 novel coronavirus (2019-nCoV) infection have gradually appeared in China. The clinical features include fever, dry cough, shortness of breath, and inflammatory changes in the lungs. In some patients, the lung lesions are progressively exacerbated, rapidly progressing to severe conditions, and the prognosis is poor. To standardize the clinical diagnosis and treatment of severe and critical novel coronavirus pneumonia (NCP) patients, improve the success rate of clinical treatment, and reduce the mortality rate, based on the "National Coronary Health Commission's NCP Diagnosis and Treatment Program (Trial Implementation of the Revised Fifth Edition)", the expert team of Peking Union Medical College Hospital proposed and formulated a diagnosis and treatment program, which we called PUMCH criterion. The focus of this criterion is on the diagnosis and treatment of severe and critically ill patients, and the content is more specific and is conducive to clinical implementation, which origin form clinical NCP treatment experience of the frontline team of PUMCH.

1. Applicable people

In line with the National Coronary Health Commission's new coronavirus diagnosis and treatment program (trial version of the fifth trial), clinically confirmed cases diagnosed as

"severe" and "critical" are as follows[1].

1.1 Severe type

Cases with the severe type meet any of the following criteria:

(1) Respiratory distress, RR ≥30 times/minute.

(2) In the resting state, the oxygen saturation is ≤93%.

(3) Arterial blood oxygen partial pressure (PaO2)/oxygen concentration (FiO2) ≤300 mmHg (1 mmHg = 0.133 kPa).

Those who have not reached the abovementioned severe diagnostic criteria but whose lung imaging shows multilobular lesions or lesion progression> 50% within 48 hours are recommended to be treated as severe patients.

The abovementioned severe diagnostic criteria have not been reached, but people who have been using hormone immunosuppressants for a long time, such as elderly individuals or individuals with diabetes, coronary heart disease, basic lung diseases, rheumatoid immune disease, or hematological tumors, and those after transplantation, should be closely monitored for the progress of the disease and, if necessary, they can be diagnosed as severe patients.

1.2 Critical type

Cases of the critical type meet one of the following conditions:

(1) Occurrence of respiratory failure that requires mechanical ventilation.

(2) Occurrence of shock.

(3) Combined organ failure requiring ICU monitoring and treatment. The diagnostic criteria of organ dysfunction was refered to Sequential Organ Failure Assessment(SOFA) scoring system [2]. In devising the score, the participants of the conference decided to limit to six the number of systems studied: respiratory, coagulation, hepatic, cardiovascular, central nervous system, and renal. A score

of 0 is given for normal function through to 4 for most abnormal, and the worst values on each day are recorded.

2. 2019-nCoV diagnosis and treatment of severe and critically ill patients

2.1Condition monitoring and clinical warning indicators

Severe and critical cases require the assessment of vital signs and clinical routine organ function. Routine blood monitoring, routine urine tests, biochemical indicators [liver enzyme (ATLandAST), myocardial enzyme(cTnI, BNP,andNT-pro-BNP), renal function (Scr andBUN), etc.], coagulation function, arterial blood gas analysis, and chest imaging should be performed according to the condition.

In addition, changes in the following indicators are helpful for assessing disease progression, and poor prognosis and can be tested according to the actual situation:

(1) Progressive decrease in peripheral blood lymphocyte count.

(2) B lymphocytes in lymphocyte count are significantly reduced, and CD4 and CD8 T cells continue to decline[3].

(3) Peripheral blood inflammatory factors such as C-reactive protein are progressively increased.

(4) Those who have these conditions should undergo high-resolution chest CT. When high-resolution CT shows a rapid expansion of the lesion, oxygen therapy and/or mechanical ventilation support should be used.

2.2 Treatment

2.2.1Foundemental treatment: rest in bed, support treatment, ensure sufficient calories; pay attention to water and electrolyte balance to maintain internal environment stability; closely monitor

vital signs and oxygen saturation. On the basis of symptomatic treatment, active respiratory support and volume management should be implemented as early as possible, taking into account systemic multiple organ damage and preventing and treating complications.

2.2.2.2 Respiratory support[4]:

(1) Patients with mild ARDS, that is, P/F is 200~300 mmHg

1) Patients with mild ARDS should receive oxygen from a nasal cannula or mask. Promptly assess whether respiratory distress and/or hypoxemia is relieved.

2) High-flow nasal cannula (HFNC) oxygen therapy: When patients with respiratory distress and/or hypoxemia cannot be relieved after receiving standard oxygen therapy, high-flow nasal cannula oxygen therapy can be considered for 2-6 hours. If it cannot be tolerated, it can be changed to noninvasive ventilation (NIV) or invasive mechanical ventilation.

(2) Patients with mild to moderate ARDS, that is, P/F is 150~200 mmHg

Initially choose NIV treatment and observe the patient for 2 hours. If Vt ≤ 9 ml/kg, continue NIV treatment; if Vt> 12 ml/kg, stop NIV immediately, and switch to tracheal intubation for invasive mechanical ventilation. If Vt is 9~12 ml/kg, then continue to observe NIV closely for 6 h. If Vt ≤ 9 ml/kg, continue NIV. If Vt> 9 ml/kg, stop NIV and switch to tracheal intubation with invasive mechanical ventilation.(3) Patients with moderate to severe ARDS, that is, P/F is 150 mmHg

1) Mechanical ventilation strategy[5]

After successful intubation, invasive mechanical ventilation can be implemented. In accordance with ARDS invasive mechanical ventilation, a "pulmonary protective ventilation strategy" should be implemented.

2) Lung recruitment[6]

Recruitment maneuvers are often effective in mechanical ventilation patients with 2019 nCoV. For patients who need FiO_2> 50% to maintain target oxygen, the assessment of lung recruitment maneuvers is required. The assessment of lung recruitment maneuvers includes ultrasound, PV curve, EIT, etc.

3) Prone position[7]

The prone position can promote the collapse of the collapsed alveoli and improve the compliance of the respiratory system. In addition, it can also improve right heart function and reduce pulmonary arterial pressure. Prone ventilation is recommended for patients with moderate to severe ARDS. Prolonged ventilation and re-alveolar alveoli are time-dependent, so prolonged prone ventilation is recommended, and prone ventilation for patients with severe ARDS is recommended for 16-20 hours per day.

4) Invasive mechanical ventilation evacuation

The patient's condition improves after treatment. If the platform pressure is <30 cmH_2O, FiO_2 is ≤ 40%, and PEEP is ≤ 5 cmH_2O, the ventilator can be changed from the control mode to the pressure support mode. Evacuation of the invasive ventilator can be considered when the following conditions are met: (1) consciousness; (2) stable circulation, that is, no vasoactive drugs or dopamine <5 μg/(kg • min) or norepinephrine <20 μg/min; (3) ventilator conditions are pressure support ventilation, FiO_2≤ 40%, PEEP≤5 cmH_2O, and SpO_2> 95% or P/F ≥250 mmHg, 35 mmHg ≤ $PaCO_2$ ≤50 mmHg or rapid shallow breathing index [Vt (ml)/RR] ≤105.

(4) ECMO application recommendations[8]

1) ECMO startup timing

Under optimal ventilation conditions (FiO_2 ≥ 0.8, tidal volume 6 ml/kg, PEEP ≥ 10 cmH_2O), ECMO can be started if there is no contraindication and if one of the following conditions is met: a.

PaO2/FiO2 <50 mmHg for more than 3 h; b. PaO2/FiO2 <80 mmHg for more than 6 h; c. FiO2 = 1.0 and PaO2/FiO2 <100 mmHg; d. arterial pH <7.25 and PaCO2> 60 mmHg for more than 6 h, and respiratory rate> 35 times/min; e. when the respiratory frequency is> 35 times/min, the pH value is <7.2 and the plateau pressure is> 30 cmH2O; f. severe air leak syndrome; and g. combined with cardiogenic shock or cardiac arrest.

2) Contraindications for ECMO use

Combination with unrecoverable diseases; contraindications to anticoagulation; under high mechanical ventilation settings [FiO2> 0.9, P-plat> 30 cmH2O], mechanical ventilation for 7 days or more; advanced age; immunosuppression (absolute neutrophil count <400/mm3); presence of anatomical deformities or lesions of surrounding large blood vessels.

3) Choice of ECMO treatment mode:

Patients with NCP predominantly have respiratory failure and generally choose the VV ECMO model. When circulatory failure occurs, its cause should be judged to determine whether the mode of VA ECMO is needed.

2.2.3Circulatory Support

For circulatory support, follow the principle of hemodynamic therapy guided by tissue perfusion. In volume management, efforts should be made to maintain a minimum volume that meets tissue perfusion to avoid fluid overload and aggravating lung injury[9].

Patients with NCP often have significant elevations in myocardial enzymes, so heart function should be monitored closely and caretakers should be alert to cardiogenic shock [10]. Additionally, heart rate management and acute coronary events prevention should also be consideration.

Patients with NCP have severe ARDS lesions in the lungs and extremely high respiratory support conditions. They are prone to acute cor pulmonale [10]. Right heart function should be closely monitored, and actively recruited maneuvers should be performed in the lungs to reduce pulmonary circulation resistance.

2.2.4. Antiviral therapy: There is no medical evidence to support the effectiveness of existing antiviral drugs against coronavirus. Options to consider include lopinavir/ritonavir or abidol. For severe and critically ill patients, the adverse reactions of antiviral drugs and the interaction with other drugs should be fully considered and the advantages and disadvantages of their use should be weighed.

2.2.5. Human immunoglobulin: Previous studies have shown that high-dose IVIg has a certain effect and safety in acute viral infections. Consider the use of high-dose intravenous infusion of human immunoglobulin (pH 4), especially 0.25-0.5 g/kg/d for severe, especially early-stage and advanced patients; the effect of advanced treatment in critically ill patients is not clear[11].

2.2.6 Recovery plasma: There is currently insufficient medical evidence to support the use of convalescent plasma. The level of protective antibody titers in plasma should be measured.

2.2.7. Glucocorticoids: There is no medical evidence to judge the efficacy of hormones in this disease. Severe patients may use glucocorticoids at an early stage as appropriate[12]. The dose is methylprednisolone 40-80 mg qd iv, and the course of treatment is 3-5 days. The course of treatment can be appropriately extended according to the clinical and imaging performance of the patient. In critically ill patients, carefully evaluate the benefits and risks of hormones before making a decision.

2.2.8. Antibacterial and antifungal treatment: According to the clinical and imaging manifestations of the patient, if secondary infection cannot be excluded, consideration should be

given to covering possible pathogens. If clinically permitted, respiratory pathogens should be actively monitored for targeted anti-infective treatment.

2.2.9. Other organ-function-oriented severe treatment: Because the direct damage of the virus itself and sequential organ damage that can occur after severe hypoxia, including in the brain, kidney, and digestive tract, as well as coagulation damage are common, during the treatment, we should pay close attention to the damage of various organ functions, actively correct shock and hypoxia, and pay attention to the recovery and protection of organ function [13].

3. Transfer-out and discharge standards [1]

Transfer-out ICU standard:

(1) Respiratory function recovers well without the need for invasive or mechanical ventilation assistance.

(2) Stable circulation, no vasoactive drugs.

(3) No other acutely progressing organ dysfunction.

4. Discharge criteria (see guidelines of the National Health Commission) [1]

The body temperature returned to normal for more than 3 days, the respiratory symptoms improved significantly, the pulmonary imaging inflammation was significantly absorbed, and the nucleic acid detection of respiratory pathogens was negative for two consecutive times (sampling interval at least 1 day).

Patients who meet the above criteria can be released from isolation or transferred to the appropriate department for the treatment of other diseases according to their condition.

References

1. Commission NCH. NCP Diagnosis and Treatment Program (Trial Implementation of the Revised Fifth Edition). 2020.

2. Vincent JL, Moreno R, Takala J, et al. The SOFA (Sepsis-related Organ Failure Assessment) score to describe organ dysfunction/failure. On behalf of the Working Group on Sepsis-Related Problems of the European Society of Intensive Care Medicine. *Intensive Care Med* 1996; **22**(7): 707-10.

3. Li T, Qiu Z, Han Y, et al. Rapid loss of both CD4+ and CD8+ T lymphocyte subsets during the acute phase of severe acute respiratory syndrome. *Chin Med J (Engl)* 2003; **116**(7): 985-7.

4. Fan E, Del Sorbo L, Goligher EC, et al. An Official American Thoracic Society/European Society of Intensive Care Medicine/Society of Critical Care Medicine Clinical Practice Guideline: Mechanical Ventilation in Adult Patients with Acute Respiratory Distress Syndrome. *Am J Respir Crit Care Med* 2017; **195**(9): 1253-63.

5. Chiumello D, Brochard L, Marini JJ, et al. Respiratory support in patients with acute respiratory distress syndrome: an expert opinion. *Crit Care* 2017; **21**(1): 240.

6. Pelosi P, Gama de Abreu M, Rocco PR. New and conventional strategies for lung recruitment in acute respiratory distress syndrome. *Crit Care* 2010; **14**(2): 210.

7. Gattinoni L, Tognoni G, Pesenti A, et al. Effect of prone positioning on the survival of patients with acute respiratory failure. *N Engl J Med* 2001; **345**(8): 568-73.

8. Combes A, Hajage D, Capellier G, et al. Extracorporeal Membrane Oxygenation for Severe Acute Respiratory Distress Syndrome. *N Engl J Med* 2018; **378**(21): 1965-75.

9. Su LX, Liu DW. Personalized Critical Hemodynamic Therapy Concept for Shock Resuscitation. *Chin Med J (Engl)* 2018; **131**(10): 1240-3.

10. Cardinal-Fernandez P, Esteban A, Thompson BT, Lorente JA. ARDS: lessons learned from the heart. *Chest* 2015; **147**(1): 7-8.

11. Hemming VG. Use of intravenous immunoglobulins for prophylaxis or treatment of infectious diseases. *Clin Diagn Lab Immunol* 2001; **8**(5): 859-63.

12. Annane D. Glucocorticoids for ARDS: Just Do It! *Chest* 2007; **131**(4): 945-6.

13. Clinical course and outcomes of critically ill patients with SARS-CoV-2 pneumonia in Wuhan, China: a single-centered, retrospective, observational study
