# Supplementary material for: Retrospective Study of Critically Ill COVID-19 Patients With and Without Extracorporeal Membrane Oxygenation Support in Wuhan, China
Source: Front Med (Lausanne). 2021 Oct 12;8:659793. doi: 10.3389/fmed.2021.659793 (PMC8546219; doi:10.3389/fmed.2021.659793)
Supplement: Supplementary file 1 [file Data_Sheet_1.zip › 20210122-Table S5 Enrolled ECMO and non-ECMO critically ill COVID-19 patients.docx]

**Table S5. Enrolled ECMO and non-ECMO critically ill COVID-19 patients**

| **Hospitals** | ECMO (N=74) | Non-ECMO (N=94) |
| --- | --- | --- |
| Wuhan Pulmonary hospital | **18** | **9** |
| ZhongNan hospital of Wuhan University | **12** | **18** |
| Wuhan Jinyintan Hospital | **9** | **30** |
| Renmin Hospital of Wuhan University | **7** | **7** |
| Tongji Hospital Affiliated to Tongji Medical College, Huazhong University of science and technology | **6** | **8** |
| West Hospital of Wuhan Union Medical College Hospital | **6** | **0** |
| Leishenshan hospital | **5** | **6** |
| Huoshenshan hospital | **3** | **6** |
| Wuhan Asia General Hospital | **3** | **5** |
| Wuhan Red Cross Hospital（Wuhan 11^th^ Hospital） | **3** | **3** |
| Wuhan No.1 hospital (Wuhan hospital of traditional Chinese and Western Medicine) | **2** | **2** |
| Total | **74** | **94** |

All the critically ill COVID-19 pneumonia patients were treated in 11 of all the 62 authorized hospitals where ECMO support were performed.

ECMO, extracorporeal membrane oxygenation; COVID-19, coronavirus disease 2019; ARDS, acute respiratory distress syndrome.
