# Supplementary material for: Retrospective Study of Critically Ill COVID-19 Patients With and Without Extracorporeal Membrane Oxygenation Support in Wuhan, China
Source: Front Med (Lausanne). 2021 Oct 12;8:659793. doi: 10.3389/fmed.2021.659793 (PMC8546219; doi:10.3389/fmed.2021.659793)
Supplement: Supplementary file 1 [file Data_Sheet_1.zip › 20210122-Table S6 Medical staff that participated in this study.docx]

**Table S6. Medical staff that participated in this study**

| **Medical teams** | **Medical staff** |
| --- | --- |
| **Peking Union Medical College Hospital** | Shu-Yang Zhang, Xiang Zhou, Bin Du, Tai-Sheng Li, Zheng-Yin Liu, Xiao-Wei Yan, Jing-Lan Wang, Yan Qin,Huan Chen, Hong-Min Zhang, Hua Zhao, Wei Jiang, Chun-Yao Wang |
| **Beijing Institute of Respiratory Medicine, Beijing Chao-Yang Hospital** | Zhao-Hui Tong, Xu-Yan Li, Bing Sun, Hang-Yong He, Xiao Tang, Rui Wang, Chun-Yan Zhang, Shu-Qin Wang, Na Wan |
| **Northern Jiangsu People’s Hospital** | Rui-Qiang Zheng, Jiang-Quan Yu, Jun Shao, Xiao-Yan Wu, Qing-Jie Zhu，Xu-Yan Li, Ming Hu, Xiao-Feng Zhong, Min Mo, Ya-Li Chao |
| **Sichuan Academy of Medical Sciences & Sichuan Provincial People's Hospital** | Xiao-Bo Huang, Lei Deng, Rong-An Liu, Hong-Li He, Qin Yang, Shi-Ya Liu, Cheng-Qiang Deng, En-Ji Zhou, Ting Wang, Ai-Min Zhang, Yong-Jian Zhong, Ai-Min Zhang, Yong-Jian Zhong |
| **Union Hospital, Tongji Medical College, Huazhong University of Science and Technology** | You Shang, Xiao-Bo Yang, Yuan Yu, Yong-Ran Wu, Hong Liu, Xiao-Jing Zou, Hua-Qing Shu, Zhi-Qiang Pang, Hui-Ling Guo, En-Hua Hu, Bo Li |
| **Huashan Hospital Affiliated to Fudan University** | Sheng-Qing Li, Hai Zou, You-Zhi Zhang, Xian-Tao Li, Jing-Wen Xia, Jing Zhang, Xiao Ran, Yi Bian, Jie Xiong, Ming-Hao Fang |
| **China-Japan Friendship Hospital** | Qing-Yuan Zhan, Min Li, Dan Jin, Jing Sun, Jin-Gen Xia |
| **The First Hospital of China Medical University** | Ren-Yu Ding, Chao Yin, Peng Yin, Jia Jia |
| **ZhongNan hospital of Wuhan University** | Zhi-Yong Peng, Bo Hu, Shu-Han Cai, Xiao Yang, Hui Xiang, Yun Luo, Xin-Bo Ding, Chao Tian, Chao-Yang Li |
| **The Second Hospital of Jilin University** | Yong-Jie Yin, Yan-Guo Qin, Hong-Zhi Sun, Zhen-Wei Tian, Min Zhang, Bing-Jie Lai, Jing-Xiao Zhang, Mu-Chen Cui, Xin-Li Ma, Dan Cui |
| **Wuhan Pulmonary hospital** | Ming Hu, Bin-Hua Shen, Ling-Ling Wu, Yu-Mei Chen, Ya Xu, Xiao-Feng Zhong, Heng-Ming Liu, Jing Zhu, Xing Cheng |
| **Zhongda Hospital, school of medicine, Southeast University** | Hai-Bo Qiu, Chun Pan, Wei Zhang, Lu Zhang, Bin Zhang, Hong Liu, Yong-Ran Wu |
| **Wuhan Jinyintan Hospital** | Chao-Lin Huang, Ding-Yu Zhang |
