# Supplementary material for: Retrospective Study of Critically Ill COVID-19 Patients With and Without Extracorporeal Membrane Oxygenation Support in Wuhan, China
Source: Front Med (Lausanne). 2021 Oct 12;8:659793. doi: 10.3389/fmed.2021.659793 (PMC8546219; doi:10.3389/fmed.2021.659793)
Supplement: Supplementary file 1 [file Data_Sheet_1.zip › 20210122-Table S7 Comparison between survivors and non-survivors of all the enrolled patients.docx]

**Table S7. Comparison between survivors and non-survivors of all the enrolled patients**

|  | | Total | | Survivors (n=35) | Non-survivors (n=133) | | | P |  |  |
| --- | --- | --- | --- | --- | --- | --- | --- | --- | --- | --- |
| **Baseline characteristics** | | | | | | | | | |  |
| Gender（Male%） | | 105(62.5%) | | 21（60%） | 84（63.2%） | | | 0.731 |  |  |
| Age（years） | | 63(55-71) | | 56(45-63) | 64(58-73) | | | 0.001 |  |  |
| comorbidities  Hypertension  Diabetes mellitus  Cardiovascular disease  Chronic pulmonary disease  Chronic kidney disease  Chronic liver disease  Digestive disease  Cerebrovascular disease  Autoimmune and hematopathy  Solid tumor  Time from onset to admission(days)  SOFA score | | 78(46.4%)  40(23.8%)  36(21.4%)  8(4.8%)  12(7.1%)  13(7.7%)  5(3%)  13(7.7%)  4(2.4%)  9(5.4%)  11(7-18.5)  8 (7-9) | | 17（48.6%）  9（25.7%）  9（25.7%）  1（2.9%）  4（11.4%）  1（2.9%）  0  3（8.6%）  0  0  13(7.5-20)  8 (7-10) | 61（45.9%）  31（23.3%）  27（20.3%）  7（5.3%）  8（6%）  12（9%）  5（3.8%）  10（7.5%）  4（3%）  9（6.8%）  10(6-17.7)  8 (6-9) | | | 0.775  0.766  0.487  0.552  0.269  0.225  0.244  0.836  0.299  0.114  0.855  0.328 |  |  |
| **Vital signs at admission** | | | | | | | | | |  |
| Heart rate (beats per minute)  Temperature (℃)  Systolic blood pressure(mmHg)  Diastolic blood pressure(mmHg)  Respiratory Rate (beats per minute) | | 93±19  36.9±0.9  125±21  74±13  23±6 | | 98±20  36.9±0.9  124±21  75±14  24±6 | 91±19  36.9±0.9  125±21  73±12  22±5 | | | 0.076  0.773  0.756  0.365  0.207 |  |  |
| **Laboratory results at admission** | | | | | | | | | |  |
| White Blood Cell (*109/L)  Neutrophil (*109/L)  Lymphocyte (cells/dL)  Lactate (mmol/l)  Platelet (*109/L)  Total Bilirubin (umol/L)  Creatinine (umol/L)  High sensitivity C-reactive protein (mg/L)  Erythrocyte Sedimentation Rate (mm/H)  Ferritin (ng/ml)  Procalcitonin(ng/ml)  (1,3) - β - D-glucan (pg/ml)  Interleukin-6 (pg/ml)  Interleukin-8 (pg/ml) | | 10.8±6.3  9.59±5.84  710±490  2.8±2.3  129.8±105.7  17.6±12.5  89.7±82  83±72.7  57.1±35.3  1092±1139  2.8±7.9  47.6±38.4  172.9±484.9  49.6±42.3 | | 12.6±7.3  9.75±6.0  844±595  2.3±1.5  188.8±86  16.7±10.1  99.9±148.7  61.8±52.7  62.3±33.6  838.7±946.9  1.5±3.7  44.2±33.9  345±592  53.2±51.7 | 10.4±5.9  9.54±5.8  677±461  2.9±2.5  177.5±110  17.8±13.0  87.2±55.4  89±77  55.5±35.9  1193±1211  3.1±8.7  48.4±39.8  125±443  47.9±40.1 | | | 0.076  0.868  0.096  0.415  0.61  0.691  0.487  0.098  0.48  0.414  0.336  0.785  0.065  0.826 |  |  |
| **Respiratory parameters** | | | | | | | | | |  |
| **At admission**  PaO2 (mmHg)  FiO2 (%)  PaCO2 (mmHg)  PH value  **Before intubation**  PaO2 (mmHg)  PaCO2 (mmHg) | | 81.5±52.5  65.5±23.5  42.5±18.7  7.33±0.42  71.9±40.3  46.9±21.1 | | 97.9±83  65.7±20.3  45.9±17.1  7.18±0.6  88.3±62.0  44.3±17 | 77.8±42.5  65.4±24.5  41.2±19.1  7.37±0.4  69.2±35.3  47.4±21.8 | | | 0.112  0.963  0.36  0.071  0.13  0.64 |  |  |
| Time from severe ARDS to intubation(days) | | 1(1-4) | | 1(1-2) | 1(1-5) | | | 0.9 |  |  |
| **Treatment strategies** | | | | | | | | | |  |
| ECMO application | | 74（44%） | | 21（60%） | 53（39.8%） | | | 0.033 |  |  |
| Transferring with ECMO | | 54(32.1%) | | 14（40%） | 40（30.1%） | | | 0.263 |  |  |
| Vasoactive drugs | | 148(88.1%) | | 29(82.9%) | 120（90.2%） | | | 0.221 |  |  |
| Anti-viral drugs | | 79(47%) | | 13(37.1%) | 66（49.6%） | | | 0.188 |  |  |
| Cortical steroids | | 142(84.5%) | | 30(85.7%) | 112（84.2%） | | | 0.827 |  |  |
| Tocilizumab | | 7(4.2%) | | 2（5.7%） | 5（3.8%） | | | 0.607 |  |  |
| Prone position | | 49(29.2%) | | 12(34.3%) | 37（27.8%） | | | 0.454 |  |  |
| **Prognosis related parameters** | | | | | | | | | |  |
| Co-infection of bacteria  ICU stays (days) | | 60 (35.7%)  18(10-30.5) | | 14 (40%)  25.5(17.5-40) | 46 (34.6%)  17(9-30) | | | 0.552  0.001 |  |  |
| Hospital stays (days) | 27 (12-39.7) | | 47 (25-73) | | | 21 (12-33) | <0.0001 | | | |

PaO2, partial pressure of oxygen; FiO2, fraction of inspired oxygen; PaCO2, partial pressure of carbon dioxide; ARDS, acute respiratory distress syndrome; ECMO, extracorporeal membrane oxygenation; ICU, intensive care unit.
