# Supplementary material for: Retrospective Study of Critically Ill COVID-19 Patients With and Without Extracorporeal Membrane Oxygenation Support in Wuhan, China
Source: Front Med (Lausanne). 2021 Oct 12;8:659793. doi: 10.3389/fmed.2021.659793 (PMC8546219; doi:10.3389/fmed.2021.659793)
Supplement: Supplementary file 1 [file Data_Sheet_1.zip › 20210122-Table S8 Multivariate regression of variables affecting in-hospital mortality of all patients.docx]

**Table S8. Multivariate regression of variables affecting in-hospital mortality of all patients**

|  | **SE** | **Wald** | **OR** | **95%CI** | ***P* value** |
| --- | --- | --- | --- | --- | --- |
| **Age** | 0.023 | 3.79 | 1.05 | 1,1.09 | 0.051 |
| **ECMO** | 0.62 | 1.82 | 0.44 | 0.13,1.46 | 0.177 |
| **IL-6** | 0 | 1.42 | 0.99 | 0.998,1 | 0.233 |
| **Lymphocyte count** | 0.53 | 0.01 | 1.04 | 0.37,2.96 | 0.938 |
| **Constant** | 1.69 | 0.27 | 0.41 |  | 0.60 |

SE, stand error; OR, Odd Ratio; CI, confidence interval; ECMO, extracorporeal membrane oxygenation.
