# Supplementary material for: Retrospective Study of Critically Ill COVID-19 Patients With and Without Extracorporeal Membrane Oxygenation Support in Wuhan, China
Source: Front Med (Lausanne). 2021 Oct 12;8:659793. doi: 10.3389/fmed.2021.659793 (PMC8546219; doi:10.3389/fmed.2021.659793)
Supplement: Supplementary file 1 [file Data_Sheet_1.zip › 20210122-Table S9 Comparison between ECMO supported patients agedΓëñ50 and >50 years old .docx]

**Table S9. Comparison between ECMO supported patients aged≤50 and >50 years old.**

|  | Total | Age≤50 (n=21) | | | | | | | | Age>50(n=53) | | | | | | | | P* | | | | | | | | |  |  |
| --- | --- | --- | --- | --- | --- | --- | --- | --- | --- | --- | --- | --- | --- | --- | --- | --- | --- | --- | --- | --- | --- | --- | --- | --- | --- | --- | --- | --- |
| **Baseline characteristics** | | | | | | | | | | | | | | | | | | | | | | | | | | | | |
| Gender（Male%） | 46（62.2%） | | 12（57.1%） | | | | 34（64.2%） | | | | | 0.575 | | | | | | | | | |  |  |  |  |  |  |  |
| comorbidities  Hypertension  Diabetes mellitus  Cardiovascular disease  Chronic pulmonary disease  Chronic kidney disease  Chronic liver disease  Digestive disease  Cerebrovascular disease  Autoimmune and hematopathy  Solid tumor  Time from onset to admission(days) | 30（40.5%）  22（29.7%）  21（28.4%）  2（2.7%）  8（10.8%）  8（10.8%）  1（1.4%）  7（9.5%）  0  2（2.7%）  10(6-17.25) | | 6（28.6%）  6（28.6%）  4（19%）  0  3（14.3%）  3（14.3%）  0  3（14.3%）  1（4.8%）  10(3-16.5) | | | | 24（45.3%）  16（30.2%）  17（32.1%）  2（3.8%）  5（9.4%）  5（9.4%）  1（1.9%）  4（7.5%）  1（1.9%）  10(6-19) | | | | | 0.187  0.891  0.262  0.367  0.545  0.545  0.526  0.372  0.492  0.823 | | | | | | | | | |  |  |  |  |  |  |  |
| **Vital signs at admission** | | | | | | | | | | | | | | | | | | | | | | | | | | | | |
| Heart rate (beats per minute)  Temperature (℃)  Systolic blood pressure(mmHg)  Diastolic blood pressure(mmHg)  Respiratory Rate (beats per minute) | 95±23  36.9±0.9  123±23  72±14  23±6 | | | 100±22  36.6±0.6  123±25  75±15  23±6 | | | | | 93±23  37.0±0.9  123±23  71±13  24±6 | | 0.261  0.086  0.95  0.32  0.561 | | | | | | | | | |  |  |  |  |  |  |  |  |
| **Laboratory results at admission** | | | | | | | | | | | | | | | | | | | | | | | | | | | | |
| White Blood Cell (*109/L)  Neutrophil (*109/L)  Lymphocyte (cells/dL)  Lactate (mmol/l)  Platelet (*109/L)  Total Bilirubin (umol/L)  Creatinine (umol/L)  High sensitivity C-reactive protein (mg/L)  Erythrocyte Sedimentation Rate (mm/H)  Ferritin (ng/ml)  Procalcitonin(ng/ml)  (1,3) - β - D-glucan (pg/ml)  Interleukin-6 (pg/ml)  Interleukin-8 (pg/ml) | 11.9±6.2  10.3±5.9  710±410  3.3±2.9  160±94  19.8±15.6  77.1±42.5  78.6±81  60.4±35.5  1668±1819  3.3±8.4  48.6±33  319.7±705  44.7±41.7 | | | 12.2±8.3  9.3±7.1  940±600  4.2±2.8  165±82  15.3±10.9  83.1±51.9  92.4±107.3  56.6±16.4  389.4±238.3  2.27±4.8  56.9±44.3  220.6±291.7  31.3±18.6 | | | | 11.8±5.2  10.7±5.5  620±280  2.9±2.9  159±98  21.2±16.6  74.4±38.2  73.1±68.7  61.5±39.5  2947.8±1817.4  3.72±9.7  37.5±0  364.1±827.8  55.4±53.8 | | | | | | | 0.786  0.391  0.004  0.301  0.821  0.259  0.501  0.426  0.792  0.014  0.528  0.491  0.549  0.425 | | | | | | | | | | |  |  |  |
| **Respiratory parameters** | | | | | | | | | | | | | | | | | | | | | | | | | | | | |
| **At admission**  PaO2 (mmHg)  FiO2 (%)  PaCO2 (mmHg)  PH value | 89.5±45.7  67.0±25.3  49.3±17.1  7.22±0.59 | | | 85.5±40.6  74.3±25.7  46.1±11.9  6.9±0.83 | | | | 90.9±47.6  64.7±25.4  50.3±18.4  7.3±0.46 | | | | | 0.715  0.395  0.451  0.028 | | | | | | | | | |  |  |  |  |  |  |
| Time from severe ARDS to intubation(days) | 1(1-6.25) | | | 1(1-1) | | | | | | 2.5(1-8) | | | | 0.004 | | | | | |  |  |  |  |  |  |  |  |  |
| **Treatment strategies** | | | | | | | | | | | | | | | | | | | | | | | | | | | | |
| Transferring with ECMO | 31（41.9%） | | | | 7（33.3%） | | | | | 24（45.3%） | | | | | | 0.348 | | | | | | | | |  |  |  |  |
| Vasoactive drugs | 73（98.6%） | | | | 21（100%） | | | | | 52（98.1%） | | | | | | 0.526 | | | | | | | | |  |  |  |  |
| Anti-viral drugs | 34（45.9%） | | | | 7（33.3%） | | | | | 27（50.9%） | | | | | | 0.171 | | | | | | | | |  |  |  |  |
| Cortical steroids | 68（91.9%） | | | | 20（95.2%） | | | | | 48（90.6%） | | | | | | 0.507 | | | | | | | | |  |  |  |  |
| Tocilizumab | 7（9.5%） | | | | 1（4.8%） | | | | | 6（11.3%） | | | | | | 0.385 | | | | | | | | |  |  |  |  |
| Prone position | 29（39.2%） | | | | 7（33.3%） | | | | | 22（41.5%） | | | | | | 0.516 | | | | | | | | |  |  |  |  |
| **ECMO related parameters** |  | | | | |  | | | |  | | | | | | | | |  | | | | | | | | |  |
| Time from intubation to ECMO initiation(days)  Time from severe ARDS to ECMOinitiate(days)  Duration of ECMO (days)  ECMO rotation (rotate per minute)  ECMO blood flow (Liter per minute)  ECMO gas flow (L/min)  ECMO FiO2（%）  Activated Partial Thromboplastin Time (s)  Day 1 after ECMO initiation  Day 3 after ECMO initiation  Day 7 after ECMO initiation  PaO2-After ECMO (mmHg)  PaCO2-After ECMO (mmHg)  Bleeding complications  Total  Gastrointestinal Bleeding  Incision bleeding  Airway Bleeding  Hemorrhagic shock  Transfusion  Total (ml)  Red Blood Cell (ml)  Co-Infection of bacteria  Total  Incision site infection  Blood stream infection  Pulmonary bacterial infection  Mechanical complication  Obstruction  Hemolysis  prolapse | 3.5(2-8.75)  7(2-14)  13(8-21)  3069±538  3.67±0.76  4.8±1.7  67.5±25.9  51.1±24.2  58.3±28.6  54.9±17.4  103.5±58.5  45.1±13.2  36(48.6%)  25(33.8%)  6(8.1%)  12(16.2%)  29(39.2%)  5053±6694  2725±2715  29(39.2%)  2(2.7%)  13(17.6%)  28(37.8%)  2(2.7%)  7(9.5%)  1(1.4%) | | | | | 4.5(2-9.5)  5(2-10.5)  10(6-17)  3037±402  3.56±0.37  5.14±2.48  69.3±23.4  49.4±29.4  56.9±40.2  49.9±12.8  100±55.6  49.6±13.7  11(52.4%)  9(42.9%)  1(4.8%)  4(19%)  8(38.1%)  4897±5454  3013±1948  9(42.9%)  2(9.5%)  6(28.6%)  9(42.9%)  0  3(14.3%)  0 | | | | 3(2-8.75)  8(2.75-17.25)  14.5(8.25-21.75)  3084±595  3.7±0.88  4.6±1.19  66.8±27.2  51.7±22.5  58.9±22.4  57.1±18.7  105±60.3  43.3±12.8  25(47.2%)  16(30.2%)  5(9.4%)  8(15.1%)  21(39.6%)  5115±7175  2628±2939  20(37.7%)  0  7(13.2%)  19(35.8%)  2(3.8%)  4(7.5%)  1(1.9%) | | | | | | | 0.792  0.29  0.314  0.775  0.442  0.535  0.766  0.766  0.831  0.212  0.836  0.159  0.686  0.299  0.507  0.677  0.903  0.908  0.639  0.684  0.023  0.117  0.575  0.367  0.372  0.526 | | | | | | |  |  |  |  |  |
| **Prognosis related parameters** |  | | | | |  | | | |  | | | | | | | | |  | | | | | | | | |  |
| Reasons for ECMO withdrawing  Planned  Complication  Death  Weaning success  Yes  No  ICU stays (days)  Hospital stays (days)  **In-hospital mortality** | 17(23%)  12(16.2%)  45(60.8%)  29(39.2%)  45(60.8%)  21（12.75-33）  32（16-45.5）  53(71.6%) | | | | | 8(38.1%)  5(23.8%)  8(38.1%)  13(61.9%)  8(38.1%)  21(11.5-35.5)  35(14.5-63.5)  9(42.9%) | | | | 9(17%)  7(13.2%)  37(69.8%)  16(30.2%)  37(69.8%)  21(13-31.5)  32(17-41.5)  44(83%) | | | | | | | | | 0.04  0.012  0.884  0.769  0.001 | | | | | | | | |  |

ECMO, extracorporeal membrane oxygenation; PaO2, partial pressure of oxygen; PaCO2, partial pressure of carbon dioxide; ARDS, acute respiratory distress syndrome; FiO2, fraction of inspired oxygen; ICU, intensive care unit.

*P value for the comparison between patients aged≤50 and >50 years old.
