# Supplementary material for: Retrospective Study of Critically Ill COVID-19 Patients With and Without Extracorporeal Membrane Oxygenation Support in Wuhan, China
Source: Front Med (Lausanne). 2021 Oct 12;8:659793. doi: 10.3389/fmed.2021.659793 (PMC8546219; doi:10.3389/fmed.2021.659793)
Supplement: Supplementary file 1 [file Data_Sheet_1.zip › 20210122-Table S10 Time distribution of the ECMO supported patients.docx]

**Table S10. Time distribution of the ECMO supported patients**

|  | ALL ECMO | Before 2-15 (n=24) | After 2-15 (n=50) | P* | |
| --- | --- | --- | --- | --- | --- |
| **Baseline characteristics** | | | | |  |
| Sex（male%） | 46 (62.2%) | 12 (50%) | 34 (68%) | 0.135 | |
| Age（years）(M,IQR) | 58（47-66） | 59.5(46.3-63.8) | 58(47-68) | 0.930 | |
| comorbidities  Hypertension  Diabetes Mellitus  Cardiovascular disease  Chronic pulmonary disease  Chronic kidney disease  Chronic liver disease  Digestive disease  Cerebral vascular disease  Autoimmune and hematopathy  Solid tumor  Time from onset to admission(days)  SOFA score | 30（40.5%）  22（29.7%）  21（28.4%）  2（2.7%）  8（10.8%）  8（10.8%）  1（1.4%）  7（9.5%）  0  2（2.7%）  10 (6-17.25)  8 (6.75-9) | 6 (25%)  7 (29.2%)  5 (20.8%)  0  0  0  1 (4.2%)  0  0  7 (3-13.8)  8(6-9) | 24 (48%)  15 (30%)  16 (32%)  2 (4%)  8 (16%)  8 (16%)  0  7 (14%)  2 (4%)  12.5 (7-20)  9(7-9.5) | 0.059  0.941  0.319  0.321  0.038  0.038  0.146  0.054  0.321  0.156  0.145 | |
| **Vital signs at admission** | | | | |  |
| Heart rate (beats per minute)  Temperature (℃)  Systolic Blood Pressure(mmHg)  Diastolic Blood Pressure(mmHg)  Respiratory Rate (beats per minute) | 95±23  36.9±0.9  123±23  72±14  23±6 | 91±19  37.0±0.8  117±30  66±14  23±8 | 97±24  36.9±0.9  126±19  75±12  24±6 | 0.335  0.879  0.129  0.007  0.866 | |
| **Laboratory results at admission** | | | | |  |
| White Blood Cell (*109/L)  Neutrophil (*109/L)  Lymphocyte (cells/uL)  Lactate (mmol/L)  Platelet (*109/L)  Total Bilirubin (umol/L)  Creatinine (umol/L)  High sensitivity C-reactive protein (mg/L)  Erythrocyte Sedimentation Rate (mm/H)  Procalcitonin (ng/ml)  (1,3) - β - D-glucan (pg/ml)  Interleukin-6 (pg/ml)  PaO2 (mmHg)  PaCO2 (mmHg)  PH value | 11.9±6.2  10.3±6.0  710±410  3.3±2.9  160±94  19.8±15.6  77.1±42.5  78.6±81.0  60.4±35.5  3.3±8.4  48.6±33.0  319.7±705.2  89.5±45.7  49.3±17.1  7.2±0.6 | 11.5±7.5  10.5±7.1  703±408  4.8±4.8  131±56  19.6±9.1  97.6±56.9  113.5±110.5  45.9±21.3  4.4±9.9  38.1±28.4  284.8±703.8  95.4±67.3  44.6±12.5  7.0±0.8 | 12.0±5.6  10.2±5.5  710±420  3.0±2.2  175±105  19.9±18.1  66.3±27.8  62.0±57.2  66.8±39.0  2.8±7.9  56.5±38.1  333.6±717.2  87.0±33.2  51.5±18.5  7.3±0.4 | 0.764  0.834  0.953  0.221  0.088  0.939  0.01  0.025  0.199  0.506  0.516  0.842  0.547  0.185  0.098 | |
| **Treatment strategies** | | | | |  |
| Inter-hospital transfer  vasoactive drugs  anti-viral drugs  cortical steroids  Tocilizumab  Prone position  Time from severe ARDS to intubation(days) | 31（41.9%）  72（97.3%）  34（45.9%）  68（91.9%）  7（9.5%）  29（39.2%）  1（1-6.25） | 7 (29.2%)  22 (91.7%)  8 (33.3%)  21 (87.5%)  0  6 (25%)  1(1-4) | 24 (48%)  50 (100%)  26 (52%)  47 (94%)  7 (14%)  23 (46%)  1(1-7) | 0.124  0.146  0.131  0.338  0.054  0.083  0.236 | |
| **ECMO related parameters** | | | | |  |
| Time from intubation to ECMO initiation(days)  Time from severe ARDS to ECMO initiate(days)  Duration of ECMO (days)  ECMO rotation (rotate per minute)  ECMO blood flow (Liter per minute)  ECMO gas flow (L/min)  ECMO FiO2（%）  Activated Partial Thromboplastin Time (s)  Day 1 after ECMO initiation  Day 3 after ECMO initiation  Day 7 after ECMO initiation  PaO2-Pre ECMO (mmHg)  PaCO2-Pre ECMO (mmHg)  PaO2-After ECMO (mmHg)  PaCO2-After ECMO (mmHg)  Bleeding complications  Total  Gastrointestinal Bleeding  Incision bleeding  Airway Bleeding  Hemorrhagic shock  Transfusion  Total (ml)  Red Blood Cell (ml)  Co-Infection of bacteria  Total  Incision site infection  Blood stream infection  Pulmonary bacterial infection  Mechanical complication  Obstruction  Hemolysis  prolapse | 3.5（2-8.8）  7（2-14）  13（8-21）  3069±538  3.7±0.8  4.8±1.7  68±26  51.1±24.2  58.3±28.6  54.9±17.4  82.1±42.1  52.5±18.0  103.5±58.5  45.0±13.2  36（48.6%）  25（33.8%）  6（8.1%）  12（16.2%）  29（39.2%）  5053±6694  2725±2715  29（39.2%）  2（2.7%）  13（17.6%）  28（37.8%）  2(2.7%)  7(9.5%)  1(1.4%) | 2(1-5)  5(1-8)  9.5(5.3-17)  3196±485  3.7±0.4  5  62±26  52.2±27.2  49.9±12.8  53.1±23.3  75.3±32.5  47.9±15.3  76.4±23.3  41.8±8.7  6 (25%)  2 (8.3%)  1 (4.2%)  3 (12.5%)  6 (25%)  2676±2218  2062±1961  4 (16.7%)  0  0  4 (16.7%)  2 (8.3%)  1 (4.2%)  0 | 5(2-11)  9.5(3.3-17.8)  14(9-22)  3015±557  3.7±0.9  4.8±1.7  70±26  50.5±23.1  61.7±32.4  55.3±16.4  83.9±44.4  53.8±18.8  108.4±61.8 45.6±13.9  30 (60%)  23 (46%)  5 (10%)  9 (18%)  23 (46%)  5913±7538  2965±2924  25 (50%)  2 (4%)  13 (26%)  24 (48%)  0  6 (12%)  1 (2%) | 0.312  0.794  0.471  0.262  0.858  0.911  0.373  0.809  0.195  0.760  0.671  0.361  0.186  0.488  0.005  0.001  0.389  0.548  0.083  0.01  0.178  0.006  0.321  0.006  0.009  0.039  0.281  0.485 | |
| **Prognosis related parameters** | | | | |  |
| Reasons for ECMO withdrawing  Planned  Complication  Death  Weaning success  Yes  No  ICU stays (days)  Hospital stays (days)  In-hospital mortality | 17（23%）  12（16.2%）  45（60.8%）  29（39.2%）  45（60.8%）  21(12.75-33)  32(16-45.5)  53 (71.6%) | 7 (29.2%)  0  17 (70.8%)  10 (41.7%)  14 (58.3%)  14.5(10-27.5)  18.5(12-32.8)  18 (75%) | 10 (20%)  12 (24%)  28 (56%)  19 (38%)  31 (62%)  23.5(16.3-34)  36.5(25.5-53)  35 (70%) | 0.031  0.762  0.115  0.016  0.655 | |

ECMO, extracorporeal membrane oxygenation; ARDS, acute respiratory distress syndrome; FiO2, fraction of inspired oxygen; PaO2, partial pressure of oxygen; PaCO2, partial pressure of carbon dioxide; ICU, intensive care unit.
