# Supplementary figures and images for: Retrospective Study of Critically Ill COVID-19 Patients With and Without Extracorporeal Membrane Oxygenation Support in Wuhan, China
Source: Front Med (Lausanne). 2021 Oct 12;8:659793. doi: 10.3389/fmed.2021.659793 (PMC8546219; doi:10.3389/fmed.2021.659793)

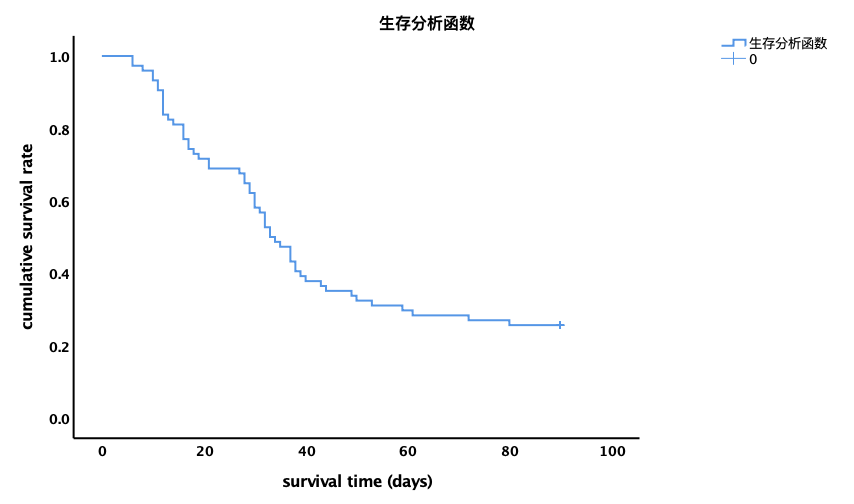

Supplement: Supplementary file 1 [file Data_Sheet_1.zip › 20210122-Figure S3 Kaplan-Meier survival analysis of the ECMO supported COVID-19 patients to 90 days post ECMO weaning.docx]

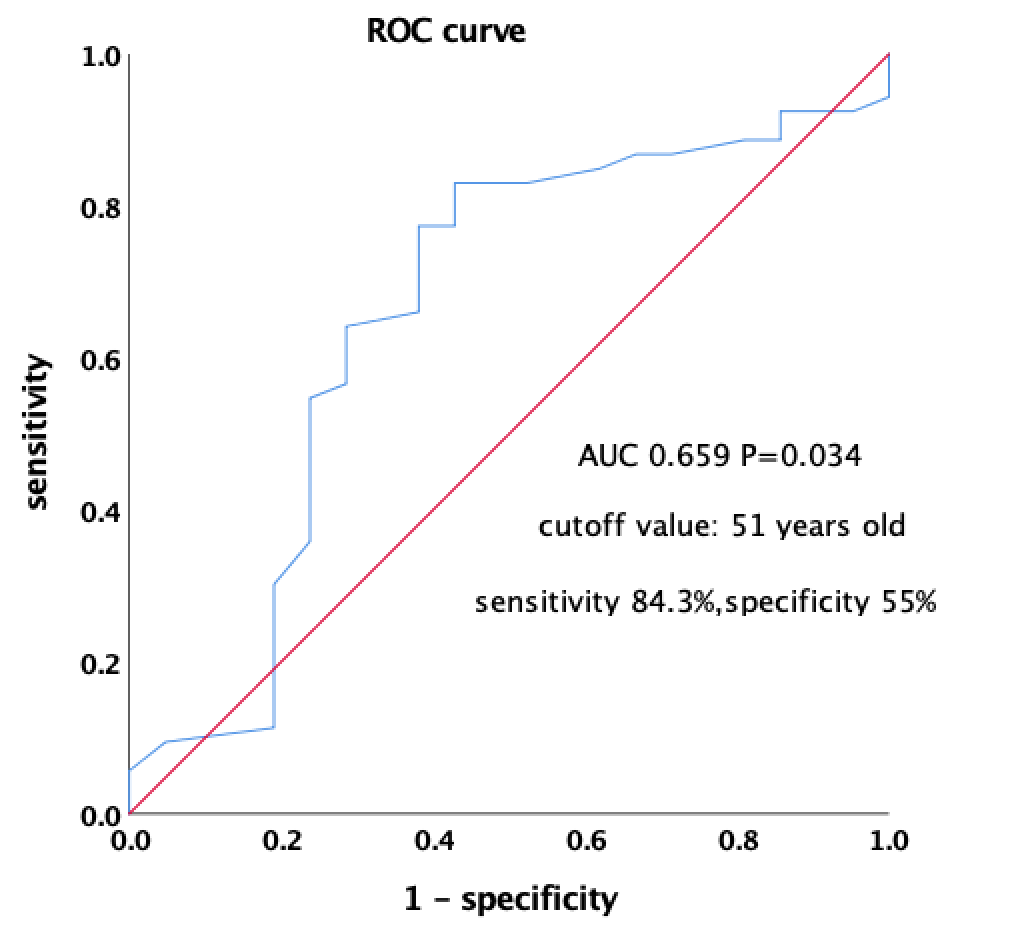

Supplement: Supplementary file 1 [file Data_Sheet_1.zip › 20210122-Figure S4 ROC curve of age for the prediction of in-hospital mortality of ECMO patients.docx]
